# Characterization of Subcellular Dynamics of Sterol Methyltransferases Clarifies Defective Cell Division in *smt2 smt3*, a C-24 Ethyl Sterol-Deficient Mutant of Arabidopsis

**DOI:** 10.3390/biom14070868

**Published:** 2024-07-19

**Authors:** Daisaku Ohta, Ayaka Fuwa, Yuka Yamaroku, Kazuki Isobe, Masatoshi Nakamoto, Atsushi Okazawa, Takumi Ogawa, Kazuo Ebine, Takashi Ueda, Pierre Mercier, Hubert Schaller

**Affiliations:** 1 Graduate School of Agriculture, Osaka Metropolitan University, 1-1 Gakuen-cho, Sakai 599-8531, Japan; ohtad@omu.ac.jp (D.O.); ogawat@omu.ac.jp (T.O.); 2 Graduate School of Life and Environmental Sciences, Osaka Prefecture University, 1-1 Gakuen-cho, Sakai 599-8531, Japan; 3 National Institute for Basic Biology, Nishigonaka 38, Myodaiji, Okazaki 444-8585, Japan; ebine@nibb.ac.jp (K.E.); tueda@nibb.ac.jp (T.U.); 4 The Graduate Institute for Advanced Studies, SOKENDAI, Nishigonaka 38, Myodaiji, Okazaki 444-8585, Japan; 5 Institute de Biologie Moléculaire des Plantes, CNRS, 12, Rue du Général Zimmer, F-67084 Strasbourg, France; pierre.mercier@ibmp-cnrs.unistra.fr (P.M.); hubert.schaller@ibmp-cnrs.unistra.fr (H.S.)

**Keywords:** Arabidopsis, C-24 ethyl sterols, cell wall, cytoskeleton, division plane, vesicle transport

## Abstract

An Arabidopsis sterol mutant, *smt2 smt3*, defective in sterolmethyltransferase2 (SMT2), exhibits severe growth abnormalities. The loss of C-24 ethyl sterols, maintaining the biosynthesis of C-24 methyl sterols and brassinosteroids, suggests specific roles of C-24 ethyl sterols. We characterized the subcellular localizations of fluorescent protein-fused sterol biosynthetic enzymes, such as SMT2-GFP, and found these enzymes in the endoplasmic reticulum during interphase and identified their movement to the division plane during cytokinesis. The mobilization of endoplasmic reticulum-localized SMT2-GFP was independent of the polarized transport of cytokinetic vesicles to the division plane. In *smt2 smt3*, SMT2-GFP moved to the abnormal division plane, and unclear cell plate ends were surrounded by hazy structures from SMT2-GFP fluorescent signals and unincorporated cellulose debris. Unusual cortical microtubule organization and impaired cytoskeletal function accompanied the failure to determine the cortical division site and division plane formation. These results indicated that both endoplasmic reticulum membrane remodeling and cytokinetic vesicle transport during cytokinesis were impaired, resulting in the defects of cell wall generation. The cell wall integrity was compromised in the daughter cells, preventing the correct determination of the subsequent cell division site. We discuss the possible roles of C-24 ethyl sterols in the interaction between the cytoskeletal network and the plasma membrane.

## 1. Introduction

Sterols play critical roles in a variety of essential functions in eukaryotic cells, such as membrane constituents and biosynthetic precursors of steroid hormones [1,2,3,4]. In plants, sterols are found as several major 24-alkyl-sterols, among which campesterol and sitosterol predominate. These sterols are mainly present in the plasma membrane (PM), and they are also found in lesser amounts in the endoplasmic reticulum (ER). The presence of sterols has been proposed in chloroplasts [5], mitochondria [6], and vacuoles [7] and is based on subcellular fractionation prior to analysis, or staining [8], although this would deserve thorough investigations. In the vast majority of seed plants including Arabidopsis, brassinosteroids, which are essential growth regulators [9], are synthesized from campesterol [10].

Arabidopsis sterol biosynthetic mutants carrying a loss of function or weak alleles of genes, implied in the cycloartenol to sitosterol pathway, exhibit severe growth inhibition, confirming the involvement of phytosterols in diverse essential functions. Sterol mutants affected in enzymes that metabolize the sterol tetracyclic nucleus from cycloartenol (4,4,14-trimethyl-9β,19-cyclo-lanostenol) to the pathway end-products (campesterol and sitosterol mainly) include *cpi1-1* carrying a transposon insertion within the cyclopropyl sterol isomerase gene [11]; *smo1* and *smo2* that harbor a deficiency of the sterol-C4-demethylation process [12,13]; *cyp51g1* that exhibits a loss-of-function obtusifoliol-14-demethylase (CYP51) [14], including *fackel/hydra2* lacking C14-reductase [15,16] and *hyd1* defected of sterol C-8,7 isomerase [17,18,19]. Defects in the methyltransferase reactions at the C-24 position in the side chain (Figure S1) also result in severe growth inhibition: *cph/smt1/orc* is defected in sterolmethyltransferase1 (SMT1) [20], and *smt2 smt3* lacks the activity of sterolmethyltransferase2 (SMT2) [21,22,23]. In *smt2 smt3* loss-of-function mutants, the biosynthesis of campesterol as the major sterol that accumulates up to 85% of the total sterols [23] (Figure S2 and Table S1) clearly shows the remarkable and specific blockage of the sitosterol (24-ethylsterols) branch of the pathway.

The growth inhibition of these sterol mutants has been primarily ascribed to the loss of the normal sterol profile, represented by the accumulation of unusual sterols derived from biosynthetic intermediates (substrate of the blocked enzymatic conversions), instead of the normal sterols (i.e., sterols present in the wild type), similar to disease states in humans [24]. The chemical structure of sterols is thought to affect the fluidity and permeability of biological membranes and the phase state (liquid order; Lo) [25,26,27,28,29,30,31]. Undoubtedly, the loss of normal sterols and the buildup of abnormal sterols must have a great impact on a wide range of cellular functions that rely on membrane properties. However, it remains unclear how a loss of the normal sterol profile results in severe growth inhibition, and, most importantly, it remains unclear how an unbalanced pathway end-sterol composition (proportions of campesterol and sitosterol) results in drastic growth inhibition. Differential contributions of phytosterols to the membrane Lo states have been studied in artificial membranes [32], as well as in the native PM of Arabidopsis [33]. Interestingly, the influence of phytosterols on membrane structuration is not equivalent for each phytosterol species, with cholesterol and campesterol showing the best ordering ability, and then sitosterol, followed by stigmasterol, in a model system [33].

One criterion for the normal (or wild-type physiological) sterol profile is the compositional ratio of C-24 methyl sterol (campesterol) to C-24 ethyl sterol (β-sitosterol and stigmasterol): the loss of the normal sterol profile, especially the strong accumulation of campesterol already mentioned above, causes strong influences on plant growth [21,34]. The difference between C-24 methyl sterols and C-24 ethyl sterols was attributed to the methylation levels at the C-24 position (Figure S1) conferred by two types of *S*-adenosyl methionine (SAM)-dependent sterolmethyltransferases, SMT1 and SMT2 (Figure S1). At the most upstream step of the sterol biosynthetic pathway, SMT1 transfers the first methyl group to the C-24 position of cycloartenol to produce 24-methylenecycloartenol. Five steps downstream of the SMT1 reaction, SMT2 adds the second methyl group onto the first methyl group at the C-24 position of 24-methylenelophenol, which was introduced by SMT1, to yield 24-ethylidenelophenol. The SMT2 reaction constitutes the branching point en route for the biosynthesis of C-24 ethyl sterols (Figure S1). In Arabidopsis, two paralogous genes, *SMT2* and *SMT3*, encode the sterolmethyltransferase2 enzyme (SMT2) that catalyzes identical reactions (Figure S1) [35].

In this study, we focused on the Arabidopsis mutant, *smt2 smt3*, which is defective in both *SMT2* and *SMT3* and exhibits severe growth inhibition. *smt2 smt3* lost only C-24 ethyl sterols, maintaining the entire pathway of C-24 methyl sterol biosynthesis (mostly campesterol) [23] and the biosynthesis of brassinosteroids (Figure S1). Note that unusual sterols derived from biosynthetic intermediates do not build up in *smt2 smt3* [23]. It has not yet been explained why a deficiency of C-24 ethyl sterols adversely affects plant growth especially after the juvenile rosette stage in ontogeny. The *smt2 smt3* cellular phenotype is similar to cytokinesis-defective mutants, such as the arrested development of the cell plate, abnormal cell shapes, multinucleated cells, and collapsed root cell files [23]. Unusual orientations of the root cortical microtubules and the root hair actin filaments in *smt2 smt3* have been reported; however, aberrations in the cytoskeletal network and defective cell division have not been addressed with respect to C-24 ethyl sterol biosynthesis [23].

To understand the biological roles of C-24 ethyl sterols, particularly during cell division, it is critical to understand the time and subcellular sites of C-24 ethyl sterol biosynthesis. In this study, we expressed a series of fluorescent proteins (GFP, mGFP, and mCherry) fused to sterol biosynthetic enzymes (Figure S1), including SMT1, SMT2, SMT3, and CYP710A1 (the C-22 desaturase at the last step of the biosynthetic pathway), under the control of endogenous promoters (Table S2). The growth inhibition of *smt2 smt3* was successfully rescued by expressing either SMT2-GFP or SMT3-mGFP but not by SMT1-GFP, indicating the inhibition was due to the defective biosynthesis of C-24 ethyl sterols. These enzymes colocalized with an ER marker and moved to the division plane during cytokinesis, indicating that the sterols were synthesized in the newly developing cell plate. Immunohistochemical studies showed that SMT2 mobilized to irregularly positioned cell plates in *smt2 smt3*. A catalytically inactive SMT2-mGFP (SMT2^D129N^-mGFP) with a point mutation [36] failed to rescue the growth inhibition accompanying a deformed endomembrane system and abnormal cell plate. Detailed studies of SMT2-GFP mobilization during cytokinesis comparing with a Qa-SNARE (KNOLLE/SYP111) [37,38], an R-SNARE (VAMP721) [39], and RABA1b, a regulator of the trans-Golgi network/early endosome (TGN/EE) trafficking [40], suggested that ER-localized SMT2-GFP mobilized to the expanding cell plate independently of Golgi-TGN-derived vesicle transport. Impaired division plane establishment was observed as early as the abnormal positioning of the preprophase band (PPB), which is a cortical microtubule array involved in preparing the future division plane [41]. This was followed by unusual phragmoplast development and aberrant cellulose deposition during cell plate development, which was visualized by Direct Red 23 staining [42]. Our results suggest that the presence of C-24 ethyl sterols is a prerequisite for the tethering of molecular components participating in the interactions between biological membranes and the cytoskeletal network.

## 2. Materials and Methods

### 2.1. Plant Materials

All experiments were conducted using *Arabidopsis thaliana* ecotype Columbia (Col-0). The plants were grown in a growth chamber maintained at 23 °C under continuous light (140–160 μmol photons m^−2^ s^−1^). After sterilization, the seeds were germinated on 0.9% (*w*/*v*) agar plates containing 1× Murashige and Skoog (MS) salts and 1% (*w*/*v*) sucrose [43]. The T-DNA insertion lines of *SMT2* (At1g20330, GABI_443_F03) and *SMT3* (At1g76090, SALK_085292) were obtained from the Arabidopsis Biological Resource Center [44]. As the homozygous double mutant *smt2 smt3* cannot set seeds, we maintained the *+/smt2;smt3/smt3* line, carrying the T-DNA insertion event within *SMT2* (*smt2*) as hemizygous and *smt3* (T-DNA insertion event within *SMT3*) as homozygous. Throughout this study, the *smt2/smt2*;*smt3/smt3* double mutant (*smt2 smt3*) was selected from the progeny of the self-pollination of *+/smt2;smt3/smt3* [23].

### 2.2. Expression Plasmids

The pSPB binary vector was generated by inserting the HindIII-EcoRI fragment of *p35S::GFP-NOST* into the pBIN Plus vector [23]. A coding sequence for mCherry was amplified by PCR using a primer set of mCherry_Fw and mCherry_Rv and inserted into BamHI-SacI double-digested pBI101 and pBI121 to express the fusion proteins with mCherry. Arabidopsis sterol biosynthetic enzymes SMT1 (AT5G13710), SMT2 (AT1G20330), SMT3 (AT1G76090), and CYP710A1 (AT2G34500) with a short-linker AAAAGGS at their C-terminus were fused to the coding sequences for the fluorescent marker proteins (GFP, mGFP, or mCherry). The fusion proteins were expressed under the control of individual promoters derived from corresponding genes in Arabidopsis. A short summary of the plasmid construction and a list of primers are shown in Figure S3 and Table S3, respectively. Briefly, for the plasmid construction of *proSMT1::SMT1-GFP*, a coding sequence region for *SMT1* (AT5G13710) together with the short-linker sequence was amplified by RT-PCR using the primer set SMT1_Fw and SMT1-linker_Rv. As an endogenous promoter region for *SMT1*, a 1629-bp fragment upstream of the *SMT1* translation initiation codon was amplified using primers pSMT1_Fw and pSMT1_Rv. For the expression of SMT2-fluorescent fusion proteins, a 2588-bp fragment was amplified from Arabidopsis genomic DNA using the primer set of pSMT2_Fw2 and SMT2_cDNA_Rv_linker. *SMT2* (AT1G20330) was encoded by a single exon. The 2588-bp fragment contained a promoter region of 1472-bp, and the entire coding region of *SMT2* (1083-bp) was used to construct the plasmids of *proSMT2::SMT2-GFP* and *proSMT2::SMT2-mCherry*. For the expression of fusion proteins with mGFP, a point mutation (^206^Ala to ^206^Lys) was introduced into *proSMT2::SMT2-GFP* using a PrimeSTAR mutagenesis basal kit (TAKARA BIO Inc., Kyoto, Japan). To express SMT3-fluorescent fusion proteins, an *SMT3* coding sequence was amplified from Arabidopsis genomic DNA using the primer sets SMT3_mCherry_Fw and SMT3_linker2_BamHI. *SMT3* (AT1G76090) is encoded by a single exon. A 1644-bp fragment was amplified using pSMT3_Fw_N and pSMT3_Rv_XbaI and used to construct the plasmids *proSMT3::SMT3-GFP* and *proSMT3::SMT3-mCherry*. The entire coding sequence for CYP710A1 (AT2G34500) was amplified from Arabidopsis genomic DNA using the primer sets At710A1Fw and At710A1_linker_RV, and the promoter region of 2021-bp was amplified using primers proAt710A1 and proAt710A1_Rv to obtain expression plasmids of *proCYP710A1::CYP710A1-GFP* and *proCYP710A1::CYP710A1-mCherry*. A point mutation (D129N) was introduced to convert ^129^Asp (GAC) to ^129^Asn (AAC) of the SMT2 protein and expressed as fusion proteins with mGFP (SMT2^D129N^-mGFP) and mCherry (SMT2^D129N^-mCherry) under the control of the endogenous *SMT2* promoter. To construct these mutant proteins, a DNA fragment encompassing the ScaI and SacI restriction sites within the SMT2 coding sequence was synthesized to introduce the D129N point mutation. The D129N point mutation is located within the putative SAM-binding site of SMT2 [26]. The nucleotide sequences of the constructed plasmids were confirmed before further experiments.

### 2.3. Plant Transformation

The Arabidopsis line (*+/smt2;smt3/smt3*) was transformed by the floral dip method using *Agrobacterium tumefaciens* strain GV3101 [45] carrying expression plasmids (Table S4). Seedlings (T_1_) were selected on MS agar plates supplemented with 100 μg/mL kanamycin. The seeds obtained from the T_1_ plants were germinated on agar plates supplemented with 5 mg/mL sulfadiazine and 200 mg/mL kanamycin, and surviving plants (T_2_) emitting fluorescence with significant intensity were identified using a fluorescence microscope (IX71; OLYMPUS Life Science, Tokyo, Japan). T_2_ seedlings were transferred to soil (Jiffy-7; Sakata Seed Co., Yokohama, Japan) to obtain T_3_ seeds. An α-tubulin-GFP expression line was generated using the expression plasmids for either the GFP-NtTubα fusion protein [46] and crossed with the *+/smt2* or the *smt3/smt3* line homozygous for *proSMT2::SMT2-mCherry*. The presence of the GFP-NtTubα expression cassette was confirmed by PCR using the primer set 35sFw (5′-TTGATGTGATATCTCCACTGACGTAAGGGA-3′) and sGFPR (5′-TGGTGCAGATGAACTTCAGGGTCAGCTT-3′). To study the cell plate localization of sterol biosynthetic enzymes, the *+/smt2;smt3/smt3* line of the homozygous for *proSMT2::SMT2-mGFP* (or *proSMT2::SMT2-mCherry*) was crossed with the Col-0 WT line expressing either GFP-KNOLLE/SYP111 [47], GFP-VAMP721 [48,49], or RFP-RABA1b [50], under the control of each endogenous promoter region. Three-day-old seedlings of the F_3_ progeny were used for further experiments. The transgenic lines expressing the fluorescent protein-fused enzymes are listed in Table S4.

### 2.4. Transformation of Tobacco BY-2 Cells

Tobacco BY-2 (*Nicotiana tabacum* L. var. Bright Yellow 2) cells were maintained in a modified Linsmaier and Skoog (LS) liquid medium supplemented with 0.2 μg/mL 2,4-D (LSD medium) [46]. Cells were cultured on a rotary shaker at 120 rpm and 25 °C in the dark. The cells were cultivated on solid culture plates made of modified LS medium containing 0.4% (*w*/*v*) gellan gum and 0.1% (*w*/*v*) MgSO4·7 H_2_O. The binary vectors for the expression of the fluorescent protein fusions were transformed into *A*. *tumefaciens* strain GV3101 [43]. The transformed *A*. *tumefaciens* cells were pre-cultured in LSD medium for 24 h. BY-2 cells (4 mL) and 100 mL of pre-cultured *A*. *tumefaciens* cells were co-incubated for 42 h at 25 °C in the dark. Cells were washed four times in 5 mL LSD medium and then plated onto solid LSD plates containing 200 μg/L kanamycin and 25 μg/mL meropenem (Sigma-Aldrich, Tokyo, Japan). Cells representing independent transformation events that appeared as calluses on the plates were individually picked and transferred onto new plates. The cells were used for the microscopic observation of fluorescent protein expression.

### 2.5. Imaging Analyses

Fluorescence detection was performed using a confocal microscope (LSM700, Carl Zeiss, Osaka Japan), and images were captured using ZEN 2011 software (Zeiss Efficient Navigation 2011). For cellulose staining, tissues were treated with 100 µg/mL Direct Red 23 (Sigma-Aldrich Japan, Tokyo, Japan) [51] for confocal microscopic analysis (LSM 700, Carl Zeiss, Germany). Clear confocal images of fixed plant tissues were obtained using ClearSee medium comprising 10% (*w*/*v*) xylitol, 15% (*w*/*v*) sodium deoxycholate, and 25% (*w*/*v*) urea [52]. The plasma membrane was observed with 5 µg/mL FM4-64 staining. Whole-mount immunolabeling experiments were performed to compare the organization of α-tubulin in plants expressing *proSMT2::SMT2-mGFP* and *proSMT2::SMT2^D129N^-mGFP*, according to a previously described method [11,23]. Antibodies were used at the following dilutions: 1:2000; mouse anti-α-tubulin IgG (Life Technologies Japan Ltd., Tokyo, Japan), 1:500; rabbit anti-GFP (Sigma-Aldrich Japan, Tokyo, Japan), 1:1000; Alexa Fluor 488 goat anti-rabbit IgG (Invitrogen), 1:500; and Alexa Fluor 568 goat anti-mouse IgG (Life Technologies Japan Ltd.). The samples were stained with 10 μg/mL 4′,6-diamidino-2-phenylindole (DAPI) for 10 min to detect nuclei in microtubule stabilizing buffer (0.1 M PIPES, 2 mM EGTA, 0.5 mM MgSO_4_, pH 7.0). The excitation and emission wavelengths were 395–540 nm for DAPI; 488–509 nm for GFP; 555–610 nm for mCherry; 515–640 nm for FM4-64; 561–633 nm for Direct Red 23; 490–525 nm for Alexa Fluor 488; 569–700 nm for Alexa Fluor 568; and 640–665 nm for Alexa Fluor 647. Fluorescence signal intensities were measured using ImageJ 1.54g software package [53]. Statistical analysis was performed using Kaleidagraph 4.1 (Synergy Software, Reading, PA, USA).

### 2.6. Sterol Analysis

Two-week-old seedlings growing on agar plates as described were flash-frozen and lyophilized. A saponification was performed on 100 mg dry weight material using 15 mL of KOH (6%) in methanol, for 2 h at 80 °C. The unsaponifiable was extracted with *n*-hexane (three times). The dried extract was acetylated according to standard procedures, and the acetylated extract was analyzed by gas chromatography as described [54,55].

## 3. Results

### 3.1. Subcellular Localizations of Sterol Biosynthetic Enzymes

In Arabidopsis, three methyltransferases (SMT1, SMT2, and SMT3) are involved in sterol biosynthesis (Figure S1). In this study, we expressed these SMTs as fluorescent protein fusions in Arabidopsis of either WT or *smt2 smt3* genotypes under the control of the promoter regions of the corresponding genes (*proSMT1::SMT1-GFP*, *proSMT2::SMT2-GFP*, *proSMT2::SMT2-mGFP*, *proSMT2::SMT2-mCherry*, and *proSMT3::SMT3-GFP*) (Table S2). The severe growth inhibition of *smt2 smt3* was rescued by expressing SMT2-GFP, SMT2-mGFP, or SMT3-GFP, but SMT1-GFP failed to restore the *smt2 smt3* phenotype (Figures S3 and S4). The successful complementation of the *smt2 smt3* mutation indicated that SMT2-GFP, SMT2-mGFP, and SMT3-GFP were functional *in planta* and confirmed the essential nature of a functional biosynthesis of C-24 ethyl sterols for plant growth. This was furthermore supported by the wild-type-like sterol profile of the seedlings expressing *proSMT2::SMT2-GFP* in the *smt2 smt3* background (Figure S2, Table S1). Conversely, SMT1-GFP was unable to supply C-24 ethyl sterols to rescue the mutant. GFP signals were prominent in the root tips (Figures S4 and S5A). The expression of SMT2-GFP was also observed in embryos (Figure S5B), and the cell division defects in *smt2 smt3* were already apparent during embryonic development (Figure S6), indicating that C-24 ethyl sterols are supplied by de novo synthesis in embryos. Next, we investigated whether a catalytically inactive SMT2 protein (SMT2^D129N^-mGFP) might be able to rescue the *smt2 smt3* phenotype. The SMT2^D129N^-mGFP protein harbors a point mutation that changes the codon Asp-129 (GAC) to Asn-129 (AAC) within the proposed site for SAM-binding [36]. The expression of SMT2^D129N^-mGFP under the control of the SMT2 promoter (*proSMT2::SMT2 ^D129N^-mGFP*) did not rescue the inhibited growth and collapsed tissue organization of *smt2 smt3* (Figures S4 and S7). In *smt2 smt3* expressing SMT2^D129N^-mGFP, the intracellular membrane network was broadly affected (Figure S7). No significant phenotypic alterations were observed in the WT plants expressing SMT2^D129N^-mGFP (Figure S4). In this study, we did not confirm the enzymatic inactivity of SMT2^D129N^-mGFP in vitro, but the failure of the genetic complementation indicated that the point mutation of D129N impaired the methyltransferase activity of SMT2, and that the growth recovery by the expression of SMT2-GFP fusion was not due to the structural role of the SMT2 protein.

**Figure 1:**
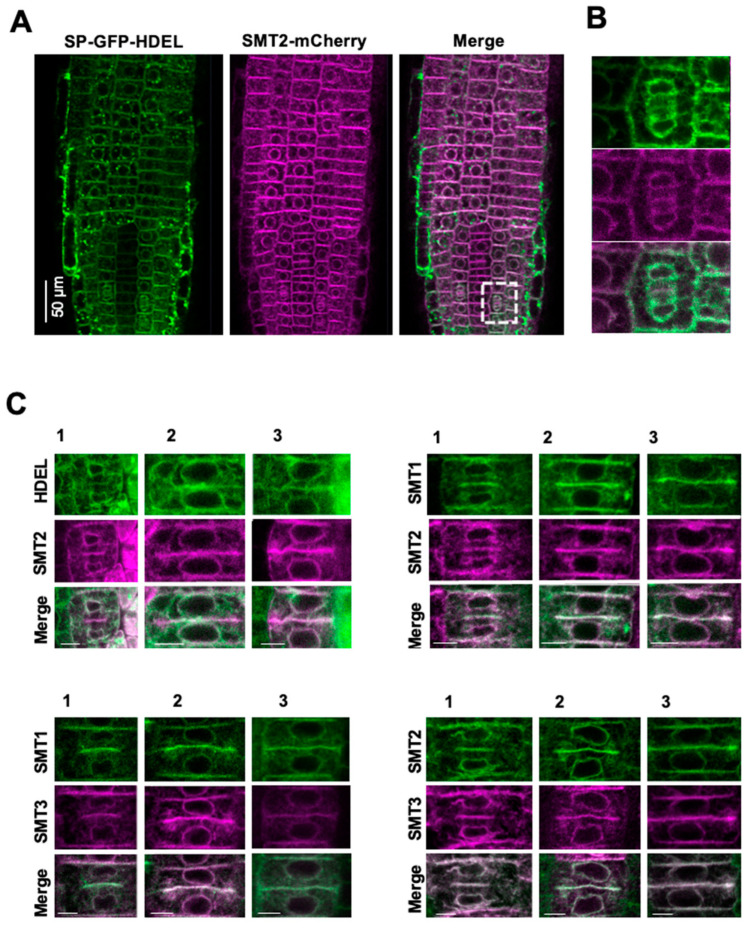
Subcellular localizations of SMT1, SMT2, and SMT3: Subcellular localizations of sterolmethyltransferases (SMT1, SMT2, and SMT3) were clarified by analyzing the colocalization with SP-GFP-HDEL (ER-targeted GFP) [56]. WT plants were transformed using *proSMT1::SMT1-GFP*, *proSMT2::SMT2-mCherry*, and *proSMT3::SMT3-mCherry*. The SMT2-mCherry expression line was crossed with the WT plants expressing SP-GFP-HDEL. SMT1-GFP and SMT2-GFP expression lines were crossed with SMT2-mCherry and SMT3-mCherry expression lines, respectively. The SMT1-GFP line was also crossed with the SMT3-mCherry line. Five-day-old seedlings were used for confocal microscopic analysis. (**A**) Colocalization of SMT2-mCherry and SP-GFP-HDEL. The region shown by the dotted square is magnified in (**B**). (**B**) Colocalization of SMT2-mCherry and SP-GFP-HDEL during cytokinesis. (**C**) Colocalization of SMT1, SMT2, and SMT3 during cytokinesis. Cells in different cytokinetic stages (1, 2, 3) are shown. Scale bars = 5 μm.

The expression patterns of SMT1-GFP, SMT2-GFP, and SMT3-GFP in the root tip region overlapped with each other, with different maximum expression zones: SMT1-GFP expression was stronger in the cortex layers, SMT2-GFP expression was observed throughout the root tip, and SMT3 expression was stronger in the cortex layers (Figure S8A). Next, we studied in detail the subcellular colocalization of SMT1, SMT2, and SMT3. The SMT1-GFP localizations overlapped with those of SMT2-mCherry and SMT3-mCherry (Figure S8B), and SMT2-mGFP co-localized with SMT3-mCherry. Interestingly, SMT2-mCherry colocalized with the ER marker SP-GFP-HDEL [56] (Figure 1A), and both were mobilized to the division plane (Figure S9). SMT1-GFP and SMT3-mCherry were also mobilized to the division plane (Figure 1).

The SMT2-mGFP fluorescent signal did not colocalize with the FM4-64 positive vesicles in the endoplasmic regions but overlapped at the division plane (Figure S10), indicating that SMT2 did not reside in the PM but was localized in the ER. In *smt2 smt3*, the catalytically inactive SMT2^D129N^-mGFP protein was also mobilized to the unusually placed division plane (Figure S7). The expression of cytochrome P450 CYP710A1-GFP was primarily found in the root epidermal cells (Figure S11A) and partially overlapped with the ER-localized SMT2-mCherry in tobacco BY2 cells (Figure S11B). These results indicated that sterol biosynthetic enzymes were localized in the ER, and that they were mobilized to the division plane (Figure S12) to supply sterols to the newly generated cell plate.

### 3.2. Endomembrane System and Cell Plate Assembly

**Figure 2:**
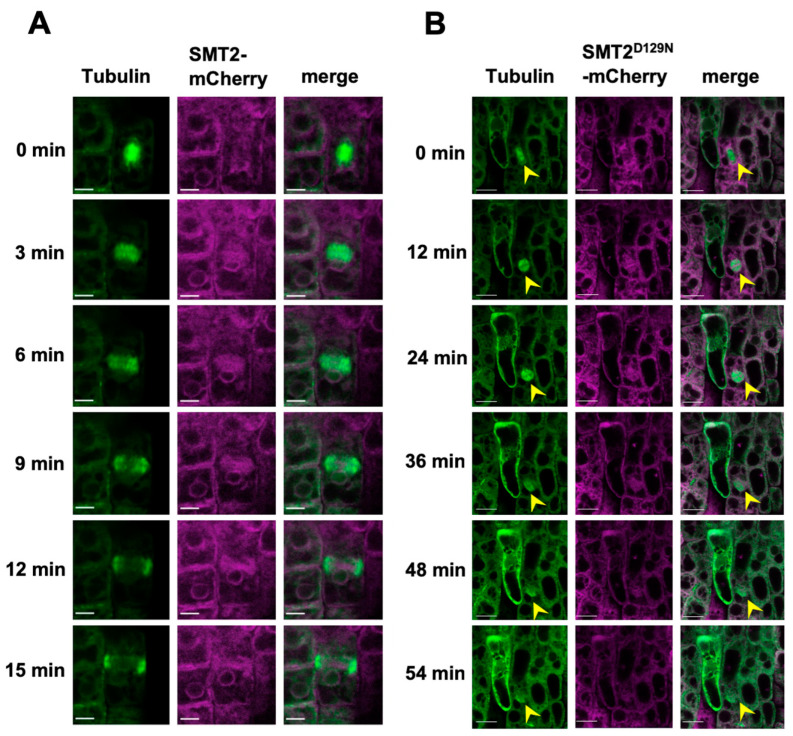
Progression of cytokinesis in WT and *smt2 smt3*: The *+/smt2;smt3/smt3* line homozygous for either *proSMT2::SMT2-mCherry* or *proSMT2::SMT2^D129N^-mCherry* was used as the pollen donor for the crossing with the WT expressing α-tubulin-GFP. From F_2_ progenies, *smt2 smt3* seedlings expressing both α-tubulin-GFP and SMT2^D129N^-mCherry were identified by screening for severe growth inhibition and collapsed tissue organizations by confocal microscopic analysis. Five-day-old seedlings were used for confocal microscopic analysis. (**A**) Cytokinesis in WT expressing α-tubulin-GFP and SMT2-mCherry. The time lapse is shown to the left. Scale bars = 5 μm. (**B**) Cytokinesis in *smt2 smt3* expressing α-tubulin-GFP and SMT2^D129N^-mCherry. Arrow heads indicate the abnormal phragmoplast. The time lapse is shown to the left. Scale bars = 5 μm.

Figure 2 compares the progression of cytokinesis in *smt2 smt3* expressing α-tubulin-GFP together with either SMT2-mCherry or SMT2^D129N^-mCherry. The cell shapes and sizes as well as the endomembrane system in *smt2 smt3* expressing SMT2^D129N^-mCherry were unusual compared to those of the SMT2-mCherry expressing line. In the SMT2-mCherry expressing line, cell division from metaphase (0 min) progressed through the formation and expansion of phragmoplasts, which reached the cortical division site (CDS) within 15 min. In the SMT2^D129N^-mCherry expressing line, cell division was arrested at the metaphase stage (12 min), and phragmoplast microtubules eventually disappeared within 54 min.

**Figure 3:**
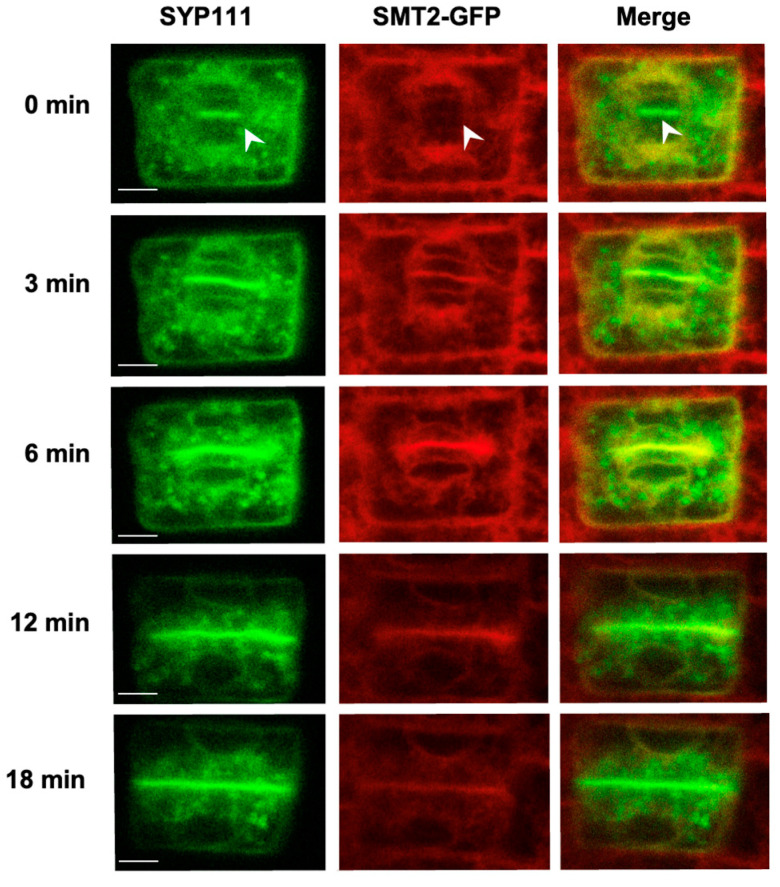
Localization of SMT2-mCherry and SYP111 during cytokinesis: The WT expressing *proSYP111::GFP-SYP111* was crossed with the *+/smt2;smt3/smt3* homozygous for *proSMT2::SMT2-mCherry*, and 5-day-old seedlings of the F_2_ progenies were used for the confocal microscopic analysis. The time lapse is shown to the left. Arrow heads indicate the division plane formation. Scale bars = 5 μm.

To clarify whether the SMT proteins were mobilized to the cell plate, we crossed *smt2 smt3* expressing SMT2-GFP or SMT2-mCherry with plants expressing GFP-KNOLLE/SYP111 [47], GFP-VAMP721 [48,49], or RFP-RABA1b [50]. Changes in the subcellular localization of these fluorescent marker proteins were monitored during cytokinesis progression. In the initial phragmoplast phase (0 min), GFP-KNOLLE/SYP111 was visible in the plane of cell division (Figure 3). SMT2-GFP was observed around the regions of nuclear membrane regeneration but did not colocalize with the GFP-KNOLLE/SYP111 vesicles (0 min) (Figure 3). As early as nuclear membrane formation was clearly observed (3 min), the colocalization of SMT2-GFP with GFP-KNOLLE/SYP111 was apparent at the division plane. The co-localization of SMT2-mCherry with VAMP721 was also observed at the division plane (Figure S13), and SMT2-GFP colocalized with RFP-RABA1b at the division plane (Figure S14). These results indicated that the ER-residing SMT2 was mobilized to the cell plate and that the mechanism responsible for SMT2 mobilization was independent of the transport of cytokinetic vesicles.

### 3.3. Cell Division Plane

**Figure 4:**
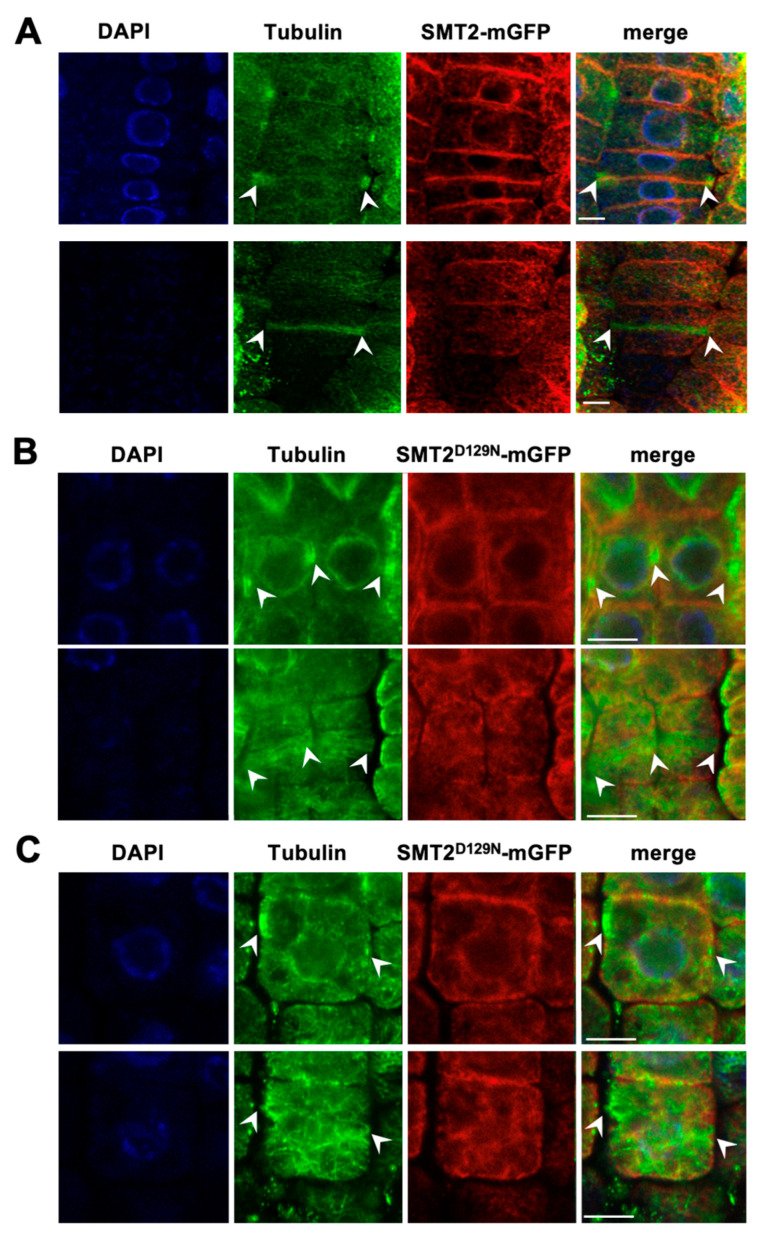
Localizations of SMT2-mGFP and microtubules during cytokinesis: The α-tubulin organizations were compared between WT expressing SMT2-mCherry (**A**) and *smt2 smt3* expressing SMT2^D128N^-mCherry (**B**,**C**). In each of (**A**) and (**B**), and (**C**), the upper panel and the lower panel shows the central part and the surface region of single cells, respectively. Antibodies were used at the following dilutions: 1:2000; mouse anti-α-tubulin IgG (Invitrogen), 1:500; rabbit anti-GFP (Sigma), 1:1000; Alexa Fluor 488 goat anti-rabbit IgG (Invitrogen), 1:500; Alexa Fluor 568 goat anti-mouse IgG (Invitrogen), 1:500; Alexa Fluor 568 goat anti-mouse IgG (Invitrogen). The samples were stained with 10 μg/mL 4′,6-diamidino-2-phenylindole (DAPI). Arrow heads indicate phragmoplasts. Scale bars = 5 μm.

We also studied SMT2 localization using immunohistochemistry. In WT expressing SMT2-mGFP, the PPB microtubules appeared horizontally at the central region of the cell, and it was visualized as a cortical ring structure on the cell cortex (Figure 4A). In the metaphase, SMT2-mGFP localization was observed around the mitotic spindle but not at the division plane. In the telophase, the SMT2-mGFP signal appeared in the developing cell plate between the late phragmoplast and the region surrounding the regenerated nuclei of the daughter cells. In *smt2 smt3*, PPB microtubules were frequently observed at odd positions in dividing cells, and cell division did not occur in the correct direction (Figures S15–S17) [23]. A typical example of perpendicular cell division was visualized in two adjacent metaphase cells (Figure S15A). Furthermore, in *smt2 smt3*, the cortical microtubules failed to form the PPB ring but displayed irregular organization, such as randomly arrayed structures, loosened assembly, and invaginations at specific positions (Figure 4B, Figures S15 and S16). In the telophase, SMT2-mGFP was observed with phragmoplast microtubules formed at unusual locations in *smt2 smt3* cells (Figure S16).

**Figure 5:**
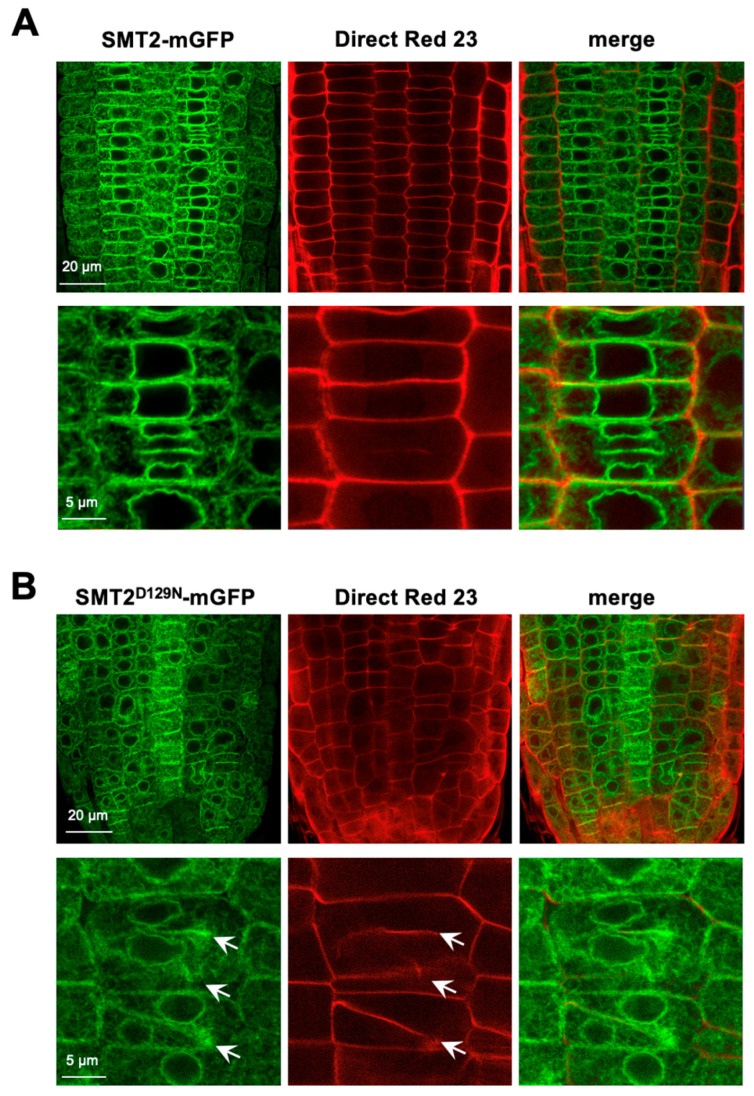
Cellulose deposition in WT and *smt2 smt3:* Cellulose deposition was compared between WT expressing SMT1-mGFP (**A**) and *smt2 smt3* expressing SMT2^D129N^-mGFP (**B**). Roots of 5-day-old seedlings were used for the staining with Direct Red 23. Arrows indicate the abnormal cell plate expansion. Scale bars are indicated in the images.

**Figure 6:**
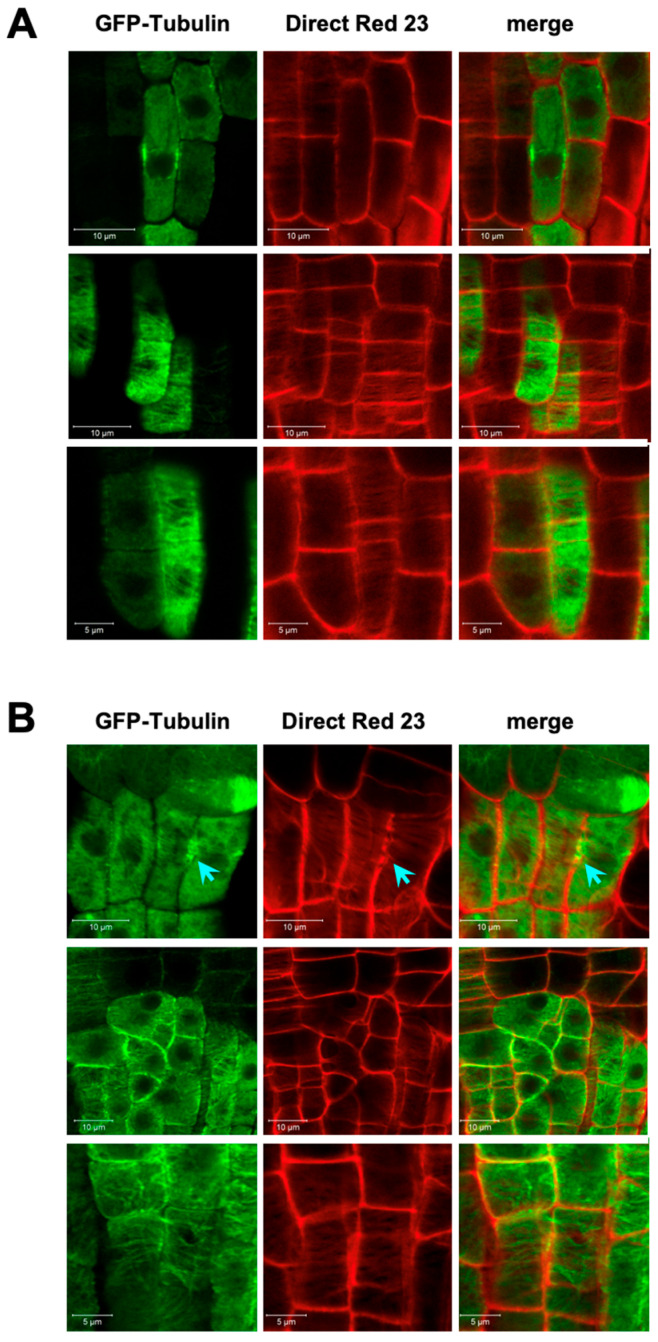
Cellulose deposition and tubulin organization in WT and *smt2 smt3*: Cellulose deposition was compared between WT (**A**) and *smt2 smt3* expressing GFP-tubulin (**B**). Roots of 5-day-old seedlings were used for the staining with Direct Red 23. Discontinuous cellulose deposition and unusual tubulin organization patterns are prominent (blue arrows). Scale bars are indicated in the images.

The failure of the correct PPB positioning in *smt2 smt3* indicated that the cell plate formation process must also have been disturbed, resulting in the disorder of the cell file organization in *smt2 smt3* (Figures S7 and S17). Cell plate formation was studied using Direct Red 23 staining to visualize cellulose deposition [42]. Clear cell shapes and solid cellulose deposition in the cell plate were visualized in WT cells expressing SMT2-mGFP (Figure 5A). In *smt2 smt3* expressing SMT2^D129N^-mGFP, the cells were unusually shaped with variable sizes and occasionally multinucleated (Figure 5B and Figure S17). Abnormalities in cellulose deposition at the developing cell plate were evident with incomplete cell plate extension, wall stubs, and unusual directions of cell division in *smt2 smt3* (Figure 5B). The halted ends of the expanding cell plates were observed as unstructured regions with scattered distributions of the fluorescent signals of Direct Red 23 and SMT2^D129N^-mGFP. In *smt2 smt3* root cells, Direct Red 23 staining also revealed indistinct or interrupted structures of the primary cell wall, and hazy areas were occasionally observed within the cells, suggesting impaired integrity of the cell wall (Figure 5B). Figure 6 compares cellulose deposition in the root tissues between WT and *smt2 smt3*, both expressing GFP-tubulin. In the irregularly oriented *smt2 smt3* cells (Figure 6B), unusual tubulin organization patterns were prominent, and discontinuous cellulose deposition was observed. Abnormal cellulose deposition at the cell surface was also apparent in *smt2 smt3* (Figure S18).

## 4. Discussion

### 4.1. Subcellular Sites of Sterol Biosynthesis

In this study, we selected *smt2 smt3* seedlings among the progeny from the self-pollination of the mutant line (*+/smt2;smt3/smt3*) because *smt2 smt3* double mutants cannot set seeds. The growth of *+/smt2;smt3/smt3* plants was supported by the supply of C-24 ethyl sterols from the remaining SMT2 activity. However, the *smt2smt3* embryos generated by *+/smt2*; *smt3/smt3* parent plants exhibited abnormal cell division (Figure S6), suggesting that C-24 ethyl sterols were not supplied by the maternal tissues of *+/smt2;smt3/smt3* but must instead be synthesized in the embryonic cells. In addition, the application of exogenous C-24 ethyl sterols to the cultured roots of *smt2 smt3*, which propagated as undifferentiated abnormal cell clumps, facilitated the development of normal roots [23]. In cultured tissues that developed new roots, the content of C-24 ethyl sterols was far below the normal level. These observations indicate that a small amount of C-24 ethyl sterols, not bulk membrane constituents, should be supplied to specific subcellular sites to promote normal development. Thus, clarification of the correct sites that require the presence of C-24 ethyl sterols in a biologically correct manner should cast light on the ambiguity of the roles of C-24 ethyl sterols. The defects in phragmoplast formation in *smt2 smt3* [23] prompted us to study in detail the adverse effects of cytokinetic events.

We demonstrated that the fluorescent protein fusions of SMT1, SMT3, SMT3, and CYP710A1 were localized in the ER, indicating that plant sterols were synthesized in the ER. These fusion proteins were all mobilized to the cell plate during cytokinesis, indicating that sterols were newly produced in the division plane. The mobilization of these ER-localized enzymes was independent of the transport of cytokinetic vesicles but colocalized with KNOLLE/SYP111, VAMP721, and RABA1b in the cell plate. The growth inhibition of *smt2 smt3* was recovered by the expression of either SMT2-mGFP or SMT3-GFP but not rescued by SMT1-GFP and the catalytically inactive SMT2^D129N^-mGFP, which were also mobilized to the cell plate, confirming that C-24 ethyl sterols are essential for plant growth (Figures S4–S7). In *smt2 smt3*, the strongly damaged structures of the entire endomembrane system, the abnormal development of the cell plate, and the failure of the division plane establishment were evident, suggesting possible structural roles of sitosterol in normal endomembrane systems (Figure 2, Figure 4 and Figure 5). Cell plate formation could be initiated, but the positions, directions, and expansion were completely disrupted, as visualized by the abnormal guidance and expansion of phragmoplasts (Figure 2, Figure 5 and Figure 6). It is possible that *smt2 smt3* retained the molecular bases involved in ER membrane remodeling and the transport of cytokinetic vesicles but could not govern such functions properly, resulting in a failure to establish the division plane and complete cell division.

### 4.2. Defected Cell Division

*smt2 smt3* formed abnormal PPBs, which were observed as randomly arrayed structures, loose assembly, and invaginations at specific positions (Figure 4 and Figures S15–S17). Thus, *smt2 smt3* cells were impaired in the organization of the cortical microtubules, resulting in the failure of CDS positioning followed by an abnormal formation of the division plane. Consequently, *smt2 smt3* could not correctly execute phragmoplast functions and failed to advance cell-plate maturation. The formation and expansion of the cell plate occasionally stopped and/or was disrupted, and the halted ends of the abnormal cell plates were surrounded by cloudy regions of the SMT2-mGFP fluorescent signal (Figure 5 and Figure 6). Direct Red 23 staining suggested the presence of cellulose debris synthesized around the cell plate but was never incorporated into the new cell wall. The filamentous structures of cellulose deposition on the WT cell surface were absent in *smt2 smt3*. It has been suggested that sitosterol, the major C-24 ethyl sterol, might be involved in the glucan polymerization initiated by CesA glucosyltransferase [57,58]; however, the current results do not provide direct evidence supporting such molecular mechanisms requiring C-24 ethyl sterols in the enzyme activities involved in cellulose biosynthesis.

We propose that the presence of C-24 ethyl sterols may be necessary in platform operation to ensure membrane protein functions, such as the cellulose synthase machinery. The hazy images of the SMT2-mGFP signals and the defective cellulose deposition at the cell plate (Figure 5B) supported the possibility that both the ER membrane rearrangement and polarized transport of the cytokinetic vesicles did not function appropriately during cell division in *smt2 smt3*. Cytokinetic vesicles from the TGN/EE network were transported along the phragmoplast microtubules to the division plane and fused with the developing cell plate. Vesicle fusion is mediated by the formation of SNARE protein complexes. It has been reported that KNOLLE/SYP111 localization to the division plane in the *cpi1-1* mutant depends on endocytosis enabled by a clathrin- and DYNAMIN-RELATED PROTEIN (DRP) 1A-dependent mechanism [59]. In *smt2 smt3*, abnormal phragmoplasts were observed with mislocalized KNOLLE/SYP111 [23], indicating that the specific loss of C-24 ethyl sterols also results in the disruption of the process of division plane establishment achieved by cytokinetic vesicle transport supported by the cytoskeletal network.

Defective cellulose deposition at the cell plate broadly affects the cell wall integrity of the daughter cells. Irregular cellulose deposition on the cell surface was also observed (Figure 6). The cell wall and cortical microtubules are suggested to be involved in sensing mechanical cues to prepare the division plane [41]. Therefore, it is possible that the impaired cell division followed by the collapsed tissue organization in *smt2 smt3* may be due to the loss of both cell wall integrity and the abnormal organization of the microtubules. However, it is yet to be clarified whether C-24 ethyl sterols can directly interact with microtubules. It has been reported that β-sitosterol stabilized microtubule assembly and showed direct binding to tubulin [60], but there is no direct evidence that such an interaction actually occurs in plant tissues.

### 4.3. Critical Roles of C-24 Ethyl Sterols

It remains unclear whether C-24 ethyl sterols have specific roles in maintaining the appropriate membrane properties that cannot be substituted by C-24 methyl sterols. Without C-24 ethyl sterols, the intracellular environment based on membrane properties may not be appropriately equipped for the execution of the normal functions of cytokinetic vesicle transport, which is supported by the cytoskeletal network [61]. We have previously reported the abnormal orientation of cortical microtubules in roots and actin filaments in root hairs in *smt2 smt3* [23].

Absolute changes in sterol composition inevitably have extensive impacts on membrane properties, such as the Lo state, particularly in the membrane microdomain [62,63]. For example, the *cpi1-1* mutant completely lost the abnormal sterol profile and defective cell plate formation [59]; the mutant could not achieve a high Lo domain in the cell-plate membrane [64]. However, such a reduction in the cell-plate Lo phase was not observed in *smt2 smt3* [23]. Tang et al. (2021) reported different PM properties in *smt2 smt3*, which was used as a sterol-depleted model, while the differential effects of C-24 ethyl sterols and C-24 methyl sterols on membrane properties remain to be clarified [64]. Borner et al. (2005) reported that sitosterol was the most abundant sterol in detergent-resistant membranes (rafts, microdomains) in Arabidopsis [65], while there was no evidence for the specific role of an individual sterol in detergent-resistant membranes. Beck et al. (2007) proposed that the alkyl group of sitosterol and stigmasterol (C-24 ethyl sterols) could reinforce the membrane cohesion properties in terms of the additional attractive van der Waals interactions with the alkyl chains of sphingolipids and phospholipids [28]; however, there was no information regarding the ratio of C-24 methyl sterol to C-24 ethyl sterol. It remains to be determined whether different states of the Lo phase might affect the localization patterns of microdomain-enriched proteins. Cytoskeletal proteins, cell wall-associated proteins, and cellular trafficking-related proteins are associated with detergent-resistant membranes [66,67,68,69]. Kang et al. (2017) reported that MICROTUBULE-ASSOCIATED PROTEIN 18 interacts with ROP2 [70], controlling its membrane localization at the root hair initiation domain [71], and is controlled by the cytoskeleton, participating in rho-of-plant (ROP) membrane localization. Of note, the strong morphogenetic defects observed for *smt2 smt3* mutants prevented an unbiased analysis of its heat stress tolerance capacity compared to wild-type rosettes. As campesterol is described in model systems as a potent membrane Lo promoter [30], it is tempting to speculate that the *smt2 smt3* capacity to buffer temperature variations is strongly abolished due to the lack of sitosterol.

Several studies have suggested that the lack of C-24 ethyl sterols strongly affects the diverse cellular functions maintained by the interactions between cytoskeletal proteins and membranes. *smt2 smt3* lacks only C-24 ethyl sterols, maintaining the C-24-methyl sterols unaffected, and the structural difference between C-24 methyl sterols and C-24 ethyl sterols is ascribed only to the presence or absence of a single methyl group in the side chain. The absence of a single methyl group in the side chain may have a significant impact on membrane properties, particularly in the membrane microdomain. It is also possible that C-24 ethyl sterols may assume unidentified essential functions such as a structural basis at the contact sites of the PM, cytoskeletal proteins, and protein factors therein. Currently, we are investigating the dynamics of proteins localized at the PM and ER contact sites, such as VAMP-associated proteins, synaptotagmins, and cytoskeletal proteins in *smt2 smt3* mutants.

In any of these cases, it is essential to clarify the subcellular localizations of C-24 ethyl sterols and their protein interactions in biological membranes and compartments. Thus far, there is no method to detect C-24 ethyl sterols in the cell specifically. A fluorescent dye, Filipin III, has been used for sterol visualization, but it binds to any sterols with 3β-OH. The development of new methods, such as the application of diazirine alkyne probes synthesized for cholesterol trafficking, is essential to elucidate the functions of C-24 ethyl sterols. Along these lines, new specific tools for our research are now emerging, such as functional alkyne phytosterols that represent powerful molecular tools to determine the cellular sites of sterol deposition and the molecular interactome of a specific sterol [72].

## Data Availability

All data are shown in this paper.

## Supplementary Materials

The following supporting information can be downloaded at: https://www.mdpi.com/article/10.3390/biom14070868/s1, Table S1. Summary of sterol biosynthetic enzymes expressed as fusion proteins with fluorescent proteins. Table S2. Sterol compositions of WT, smt2 smt3, and smt2 smt3 expressing SMT2-GFP. Table S3. Expression plasmids of sterolmethyltransferase-fluorescent protein fusions. Table S4. Primer list. Figure S1. Sterol biosynthetic pathway in Arabidopsis. Figure S2. Sterol composition of Arabidopsis seedlings analyzed by gas chromatography coupled to flame ionization detection of compounds and identification of compounds based on known retention times. Figure S3. proSMT1::SMT1-GFP and proSMT1::SMT1-mCherry; proSMT2::SMT2-GFP and proSMT2::SMT2-mCherry; proSMT3::SMT3-GFP and proSMT3::SMT3-mCherry; proCYP710A1::CYP710A1-GFP and proCYP710A1::CYP710A1-mCherry; Construction scheme of proSMT2::D129N-mGFP and proSMT2::D129N-mCherry. Figure S4. A. Genetic complementation of smt2 smt3 by expressing SMT1-GFP, SMT2-GFP, SMT2-mGFP, and SMT2D129N-mGFP; B. Root lengths of the plants expressing the fusion proteins shown in Figure S3A. Figure S5. (A)Successful complementation of smt2 smt3 by expressing SMT3-GFP. SMT1- GFP and SMT2-GFP expressing lines were shown for comparison. The fusion proteins were expressed under the control of the corresponding promoter regions. (B)  Expression of SMT2-GFP during embryonic development.Figure S6. Defected cell division in smt2 smt3 embryos. Figure S7. smt2 smt3 root tissues expressing SMT2D129N-GFP. Figure S8A. Expression patterns of SMT1-GFP, SMT2-GFP, and SMT3-GFP in root tips. Figure S8B. Colocalization of SMT1, SMT2, and SMT3. Figure S9. Colocalization of SMT2-mGFP and GFP-HDEL during cell division. Figure S10. Colocalization of SMT2-mGFP and FM4-64 at the division plane. Figure S11. Expression of CYP710A1. Figure S12. Colocalization of sterol biosynthetic enzymes at division plane. Figure S13. Colocalization of SMT2-mCherry and VAMP721. Figure S14. Colocalization of SMT2-mCherry and RABA1b. Figure S15A. Immunohistochemical analysis of the localization of SMT2-mGFP and cortical microtubules in WT. Figure S15B. Immunohistochemical analysis of the localization of SMT2-mGFP and cortical microtubules in WT. Figure S16. Colocalization of SMT2-mGFP and cortical microtubules in smt2 smt3. Figure S17 Organization of α-tubulin in smt2 smt3 plants expressing D129N- mGFP. Figure S18. Cellulose microfibril structures on the cell cortical regions of WT (A) and smt2 smt3 (B).
