# Supplementary material for: Characterization of Subcellular Dynamics of Sterol Methyltransferases Clarifies Defective Cell Division in smt2 smt3, a C-24 Ethyl Sterol-Deficient Mutant of Arabidopsis
Source: Biomolecules. 2024 Jul 19;14(7):868. doi: 10.3390/biom14070868 (PMC11275053; doi:10.3390/biom14070868)

Figure S3

proSMT1::SMT1-GFP* and *proSMT1::SMT1-mCherry

For the construction of *proSMT1::SMT1-GFP*, the coding sequence region for SMT1 (AT5G13710) was amplified by RT-PCR using the primer set SMT1_Fw and SMT1-linker_Rv. An endogenous promoter region for SMT1, a 1629-bp fragment upstream of the SMT1 translation initiation codon, was amplified using primers pSMT1_Fw and pSMT1_Rv. These PCR-amplified fragments were inserted into the HindIII – BamHI site (CaMV 35S promoter site) of the pSPB vector. For the construction of *proSMT1::SMT1-mCherry*, the HindIII – BamHI region containing the *proSMT1::SMT1* with the linker sequence of *proSMT1::SMT1-GFP* was replaced by the HindIII – BamHI fragment containing the *proSMT2::SMT2* with the linker sequence from the expression plasmid *proSMT2::SMT2-mCherry* (the construction scheme is described below).

Construction of *proSMT1::SMT1-GFP*

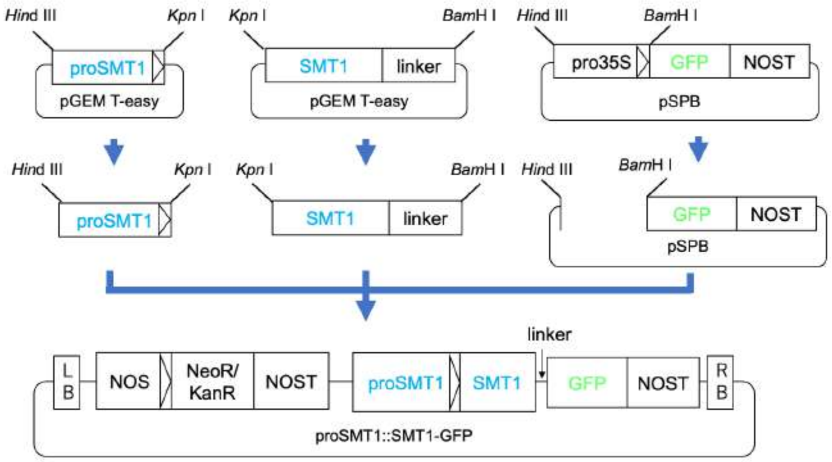

Construction of *proSMT1::SMT1-mCherry*

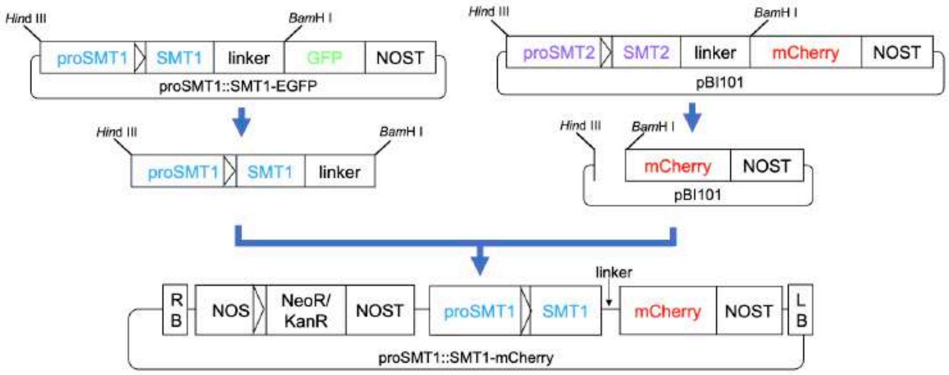

proSMT2::SMT2-GFP and proSMT2::SMT2-mCherry

SMT2 (AT1G20330) is encoded by a single exon. Therefore, for the expression of SMT2 as a fluorescent fusion protein, a 2588-bp fragment was amplified from Arabidopsis genomic DNA using the primer set of pSMT2_Fw2 and SMT2_cDNA_Rv_linker. This 2588-bp fragment contained a promoter region of 1472-bp and the entire coding region of SMT2 of 1083-bp. The 2588-bp fragment containing the sequence for *proSMT2::SMT2_linker* was double digensted with HindIII and BamHI and inserted into the corresponding site of pBI101 harboring mCherry cDNA and pSPB to obtain *proSMT2::SMT2-mCherry* and *proSMT2::SMT2-GFP*, respectively.

Construction of *proSMT2::SMT2-GFP*

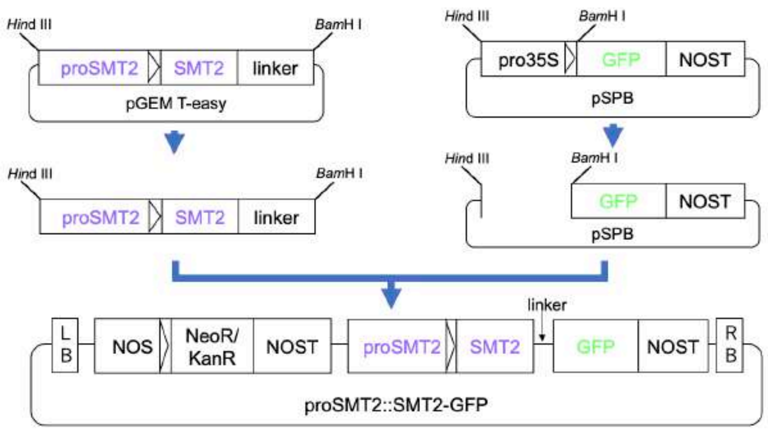

Construction of *proSMT2::SMT-mCherry*

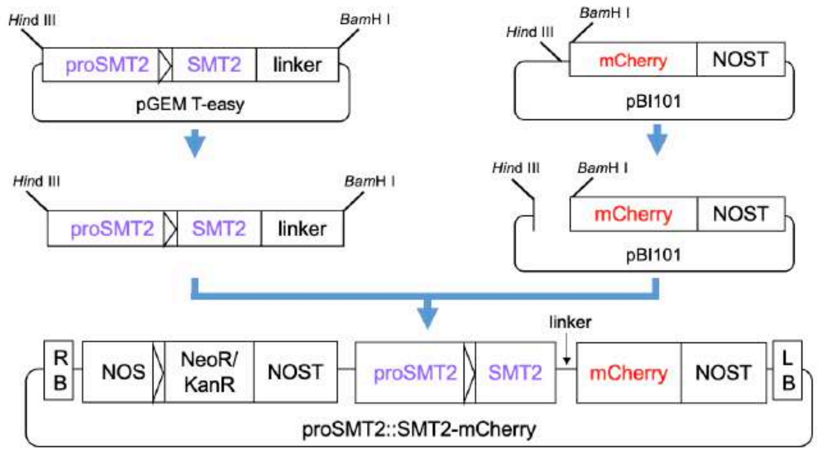

proSMT3::SMT3-GFP and proSMT3::SMT3-mCherry

SMT3 (AT1G76090) is encoded by a single exon. For the expression of SMT3-fluorescent fusion proteins, a SMT3 coding sequence was amplified from Arabidopsis genomic DNA using the primer set of SMT3_mCherry_Fw and SMT3_linker2_BamHI. A 1644-bp fragment was amplified using pSMT3_Fw_N and pSMT3_Rv_XbaI and used to construct *proSMT3::SMT3-GFP* and *proSMT3::SMT3-mCherry*.

Construction of *proSMT3::SMT3-GFP*

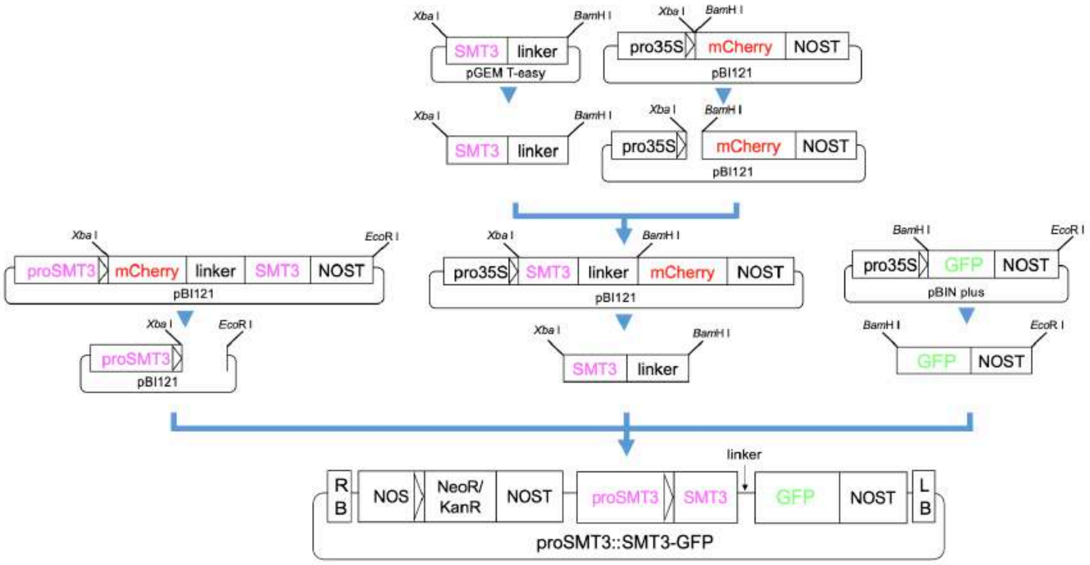

Construction of *proSMT3::SMT3-mCherry*

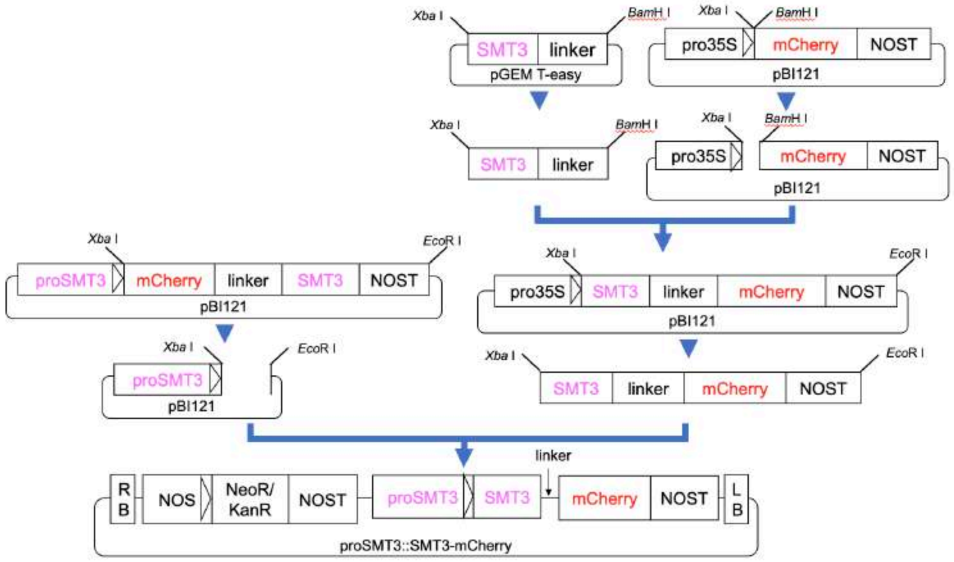

***proCYP710A1::CYP710A1-GFP* and *proCYP710A1::CYP710A1-mCherry*.**

CYP710A1 (At2g34500) is encoded by a single exon. The entire coding sequence for CYP710A1 was amplified from Arabidopsis genomic DNA using the primer set of At710A1Fw and At710A1_linker_RV, and the promoter region of 2021-bp was amplified using primers of proAt710A1 and proAt710A1 Rv. These genomic fragments were used to construct *proCYP710A1::CYP710A1-GFP* (Fig. 5S-a) and *proCYP710A1::CYP710A1-mCherry*.

Construction of *proCYP710A1::CYP710A1-GFP*

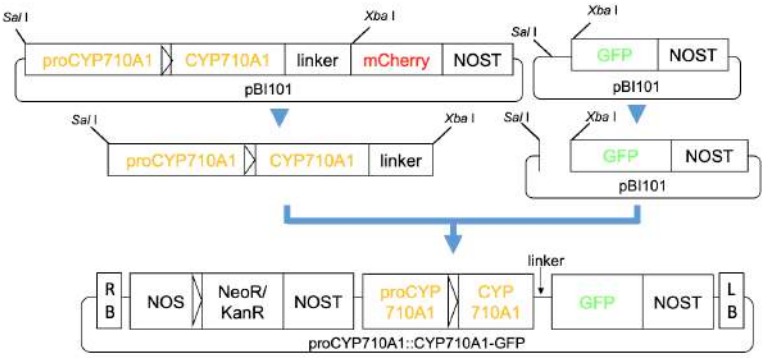

Construction of *proCYP710A1::CYP710A1-mCherry*

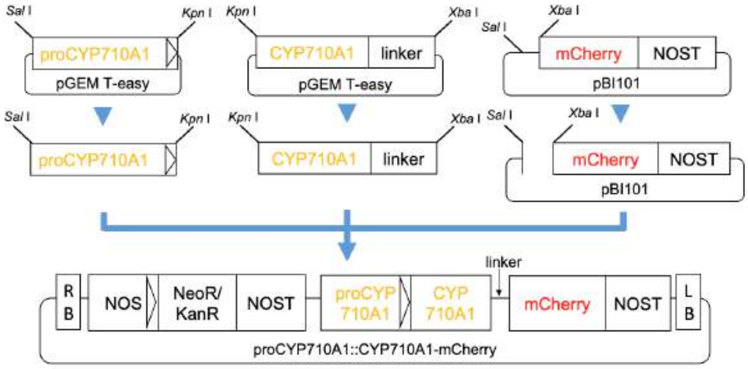

Construction scheme of *proSMT2::D129N-mGFP* and *proSMT2::D129N-mCherry*.

A point mutation (D129N) was introduced to convert ^{129}Asp to ^{129}Asn of SMT2 protein and expressed as fusion proteins with mGFP and mCherry under the control of the endogenous SMT2 promoter. A DNA fragment encompassing the ScaI and the SacI restriction sites within SMT2 coding sequence was synthesized to contain the point mutation of *D129N* and expressed as fusion proteins with mGFP and mCherry. The *D129N* is located within the putative SAM binding site of SMT2 (Kulothungan, et al. 2008).

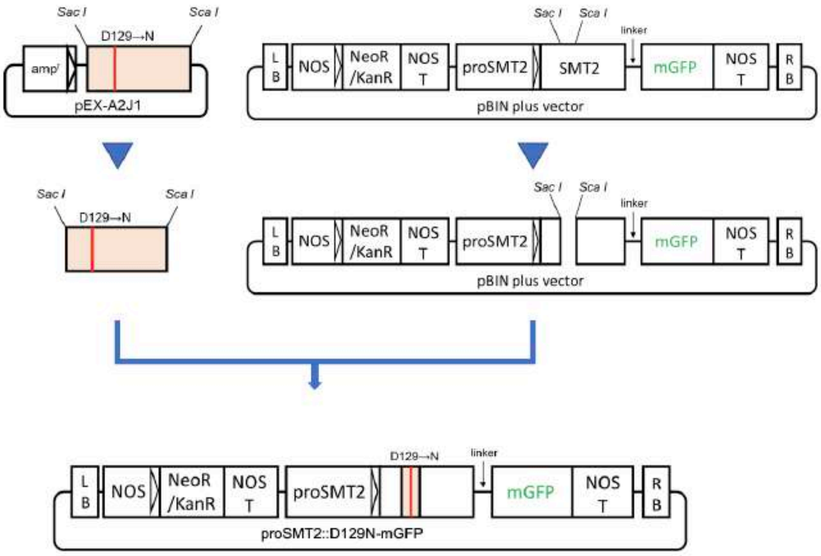

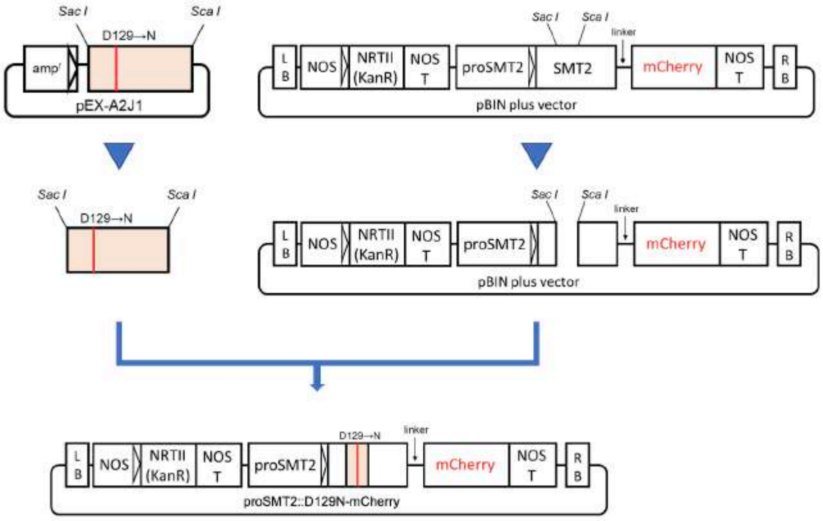

Supplement: Supplementary file 1 [file biomolecules-14-00868-s001.zip › Supplemental Figure S3.pdf]
