# Supplementary material for: Characterization of Subcellular Dynamics of Sterol Methyltransferases Clarifies Defective Cell Division in smt2 smt3, a C-24 Ethyl Sterol-Deficient Mutant of Arabidopsis
Source: Biomolecules. 2024 Jul 19;14(7):868. doi: 10.3390/biom14070868 (PMC11275053; doi:10.3390/biom14070868)
Supplement: Supplementary file 1 [file biomolecules-14-00868-s001.zip › Supplemental Figures S1 and S2.pdf]

**Figure S1**

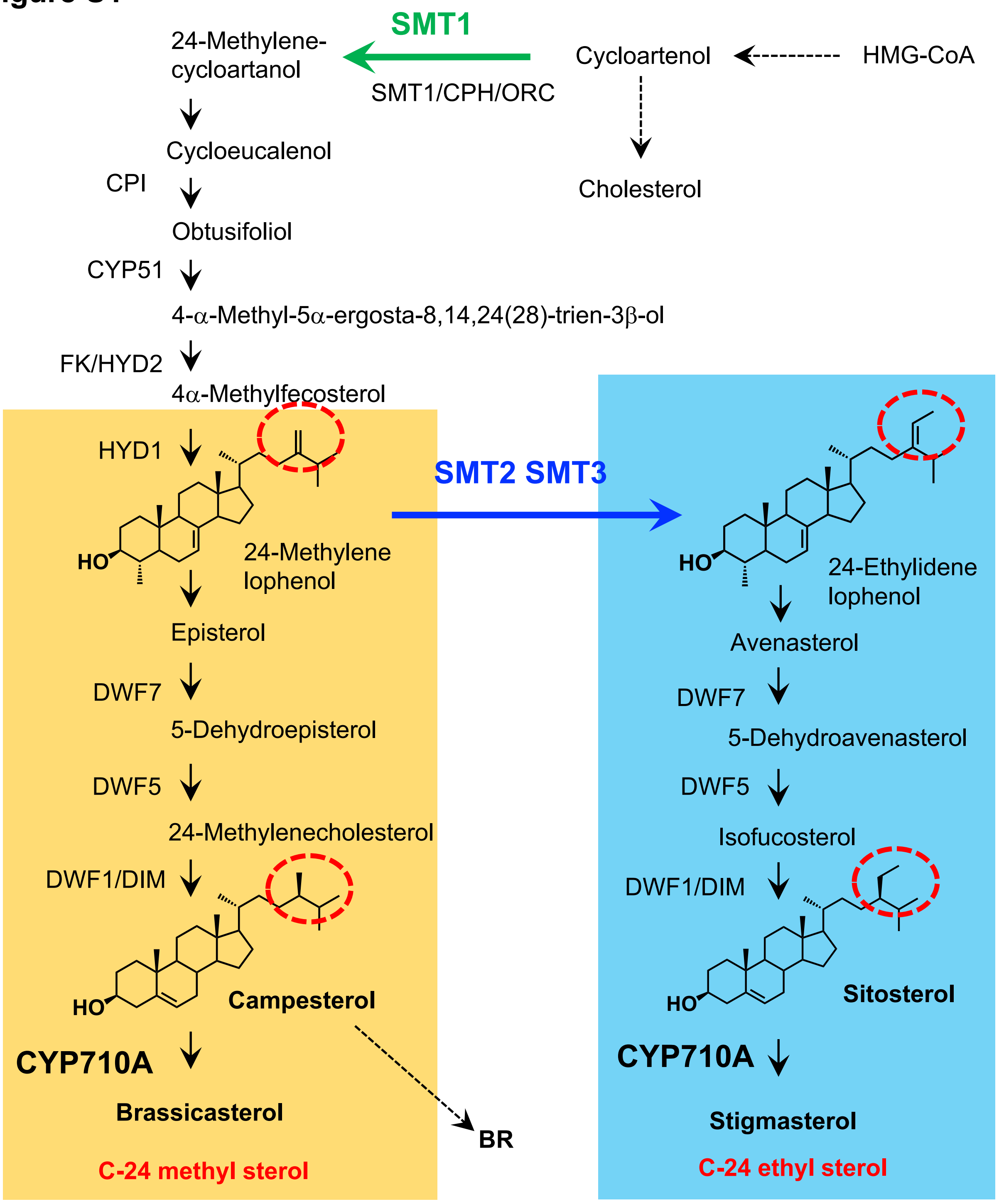


**Figure S1. Sterol biosynthetic pathway in Arabidopsis.**

The steromethyltransferase1 (SMT1) introduces the first methyl group into the C-24 position of cycloartenol, and the second methyl transfer reaction (the SMT2 reaction) is catalyzed by SMT2 and SMT3 to give the C-24 ethyl sterols (β-sitosterol and stigmasterol). The last step is the C-22 desaturase reaction by the cytochrome P450 CYP710A. The side chain structures different between C-24-methyl and C-24 ethyl sterols are indicated by dotted red circles.

# Figure S2

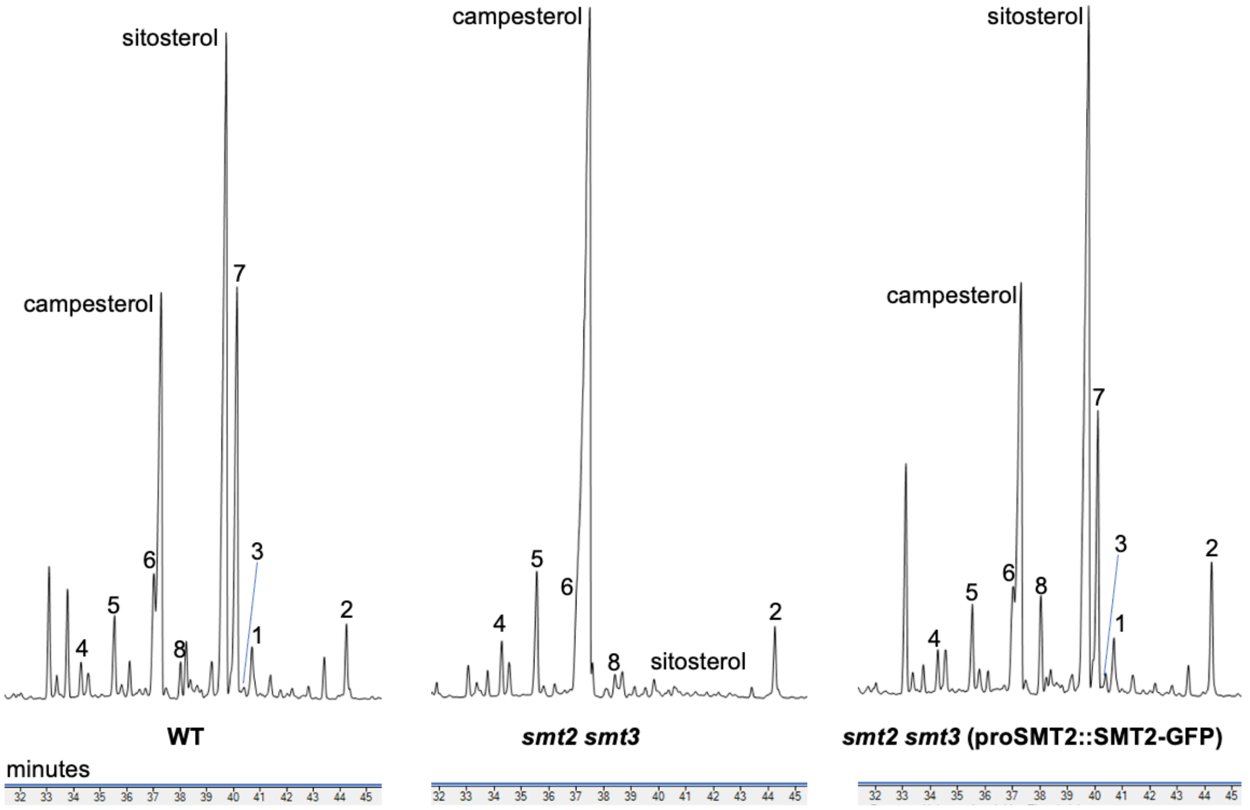


**Figure S2. Sterol composition of Arabidopsis seedlings analyzed by gas chromatography coupled to flame ionization detection of compounds and identification of compounds based on known retention times.**

Representative chromatograms of three equivalent analyses are shown. *smt2 smt3* seedlings strikingly exhibit a strongly favored 24-methylsterol (campesterol) pathway. The restauration of a wt-like profile in *smt2smt3* expressing an SMT2-GFP transgene indicates the functionality of the construct. Compounds are: 1, cycloartenol; 2, 24-methylenecycloartanol; 3, cycloeucalenol; 4, cholesterol; 5, brassicasterol; 6, 24-methylenecholesterol; 7, isofucosterol; 8, stigmasterol. Unnumbered compounds are not sterols. The table shows % of each compound for these chromatograms.
