# Supplementary material for: Characterization of Subcellular Dynamics of Sterol Methyltransferases Clarifies Defective Cell Division in smt2 smt3, a C-24 Ethyl Sterol-Deficient Mutant of Arabidopsis
Source: Biomolecules. 2024 Jul 19;14(7):868. doi: 10.3390/biom14070868 (PMC11275053; doi:10.3390/biom14070868)
Supplement: Supplementary file 1 [file biomolecules-14-00868-s001.zip › Supplemental Figures S4-S18.pdf]

Figure S4A

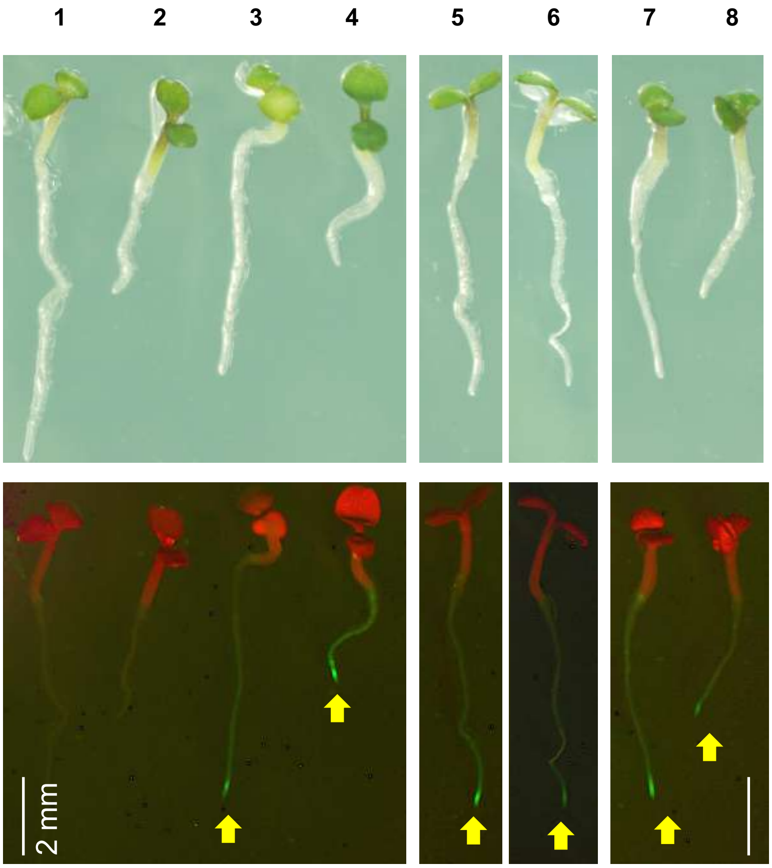

Figure S4A. Genetic complementation of *smt2 smt3* by expressing SMT1-GFP, SMT2-GFP, SMT2-mGFP, and SMT2^{D129N}-mGFP.

Yellow arrows indicate the root tips exhibiting the highest expression levels of the fluorescent protein fusions. These fusion proteins were expressed under the control of the corresponding promoter regions. 1; WT, 2; *smt2 smt3*, 3; SMT1-GFP in WT, 4; SMT1-GFP in *smt2 smt3*, 5; SMT2-mGFP in WT, 6; SMT2-mGFP in *smt2 smt3*, 7; SMT2^{D129N}-mGFP in WT, 8; SMT2^{D129N}-mGFP in *smt2 smt3*

Figure S4B

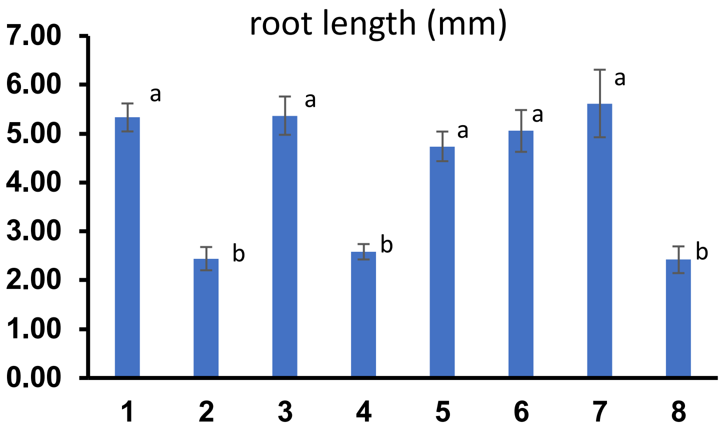

Figure S4B. Root lengths of the plants expressing the fusion proteins shown in Fig. S3A.

Different letters above the bars indicate significant difference obtained by Tukey's test (< 0.05).

The sample numbers (from 1 through 8) correspond to the same plant lines as indicated in (A).

n = 5, means ± SD. Different letters above the bars indicate the significant differences obtained by Tukey's test (< 0.05).

Figure S5

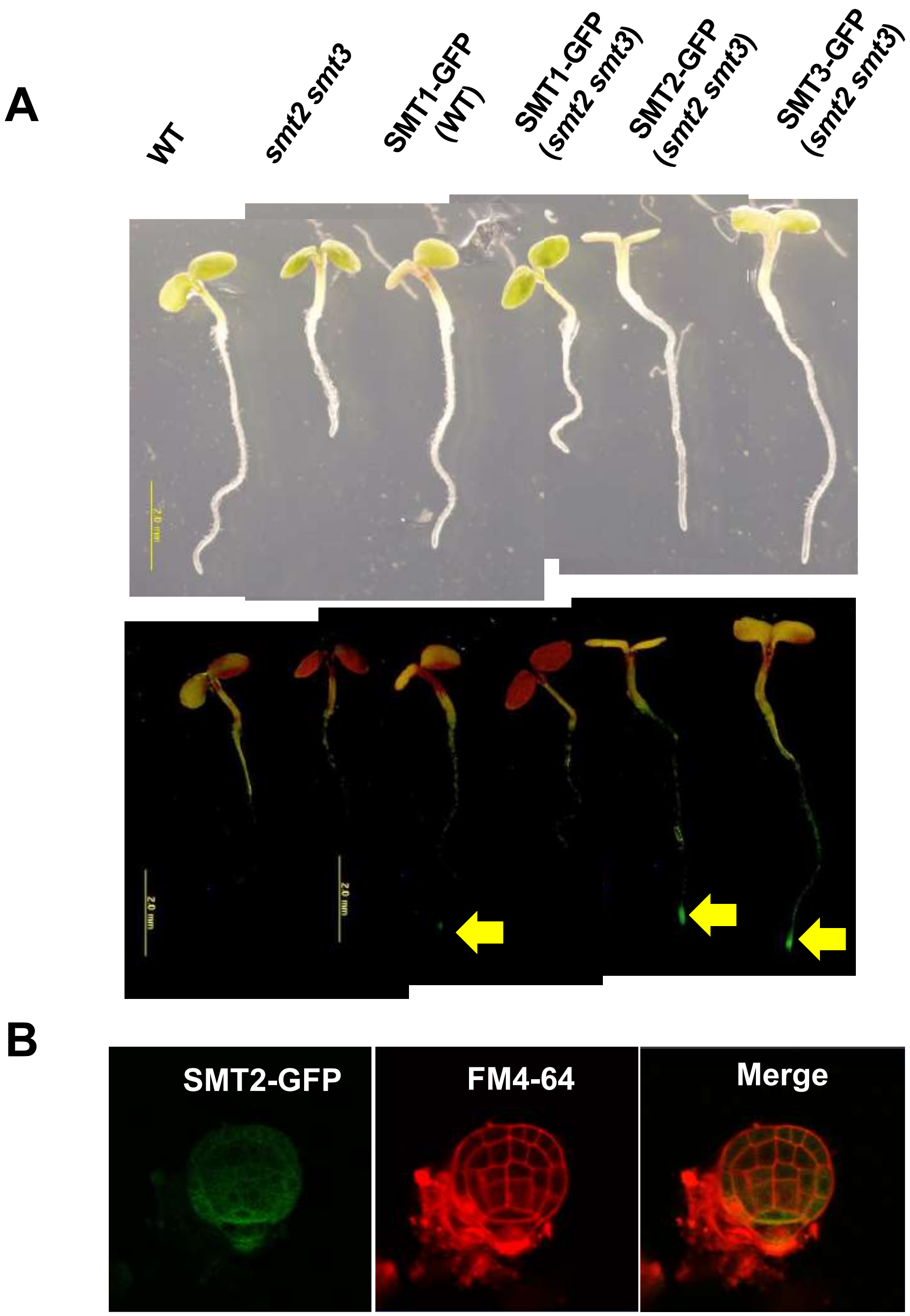

Figure S5

(A) Successful complementation of *smt2 smt3* by expressing SMT3-GFP. SMT1-GFP and SMT2-GFP expressing lines were shown for comparison. The fusion proteins were expressed under the control of the corresponding promoter regions.
(B) Expression of SMT2-GFP during embryonic development.

Figure S6

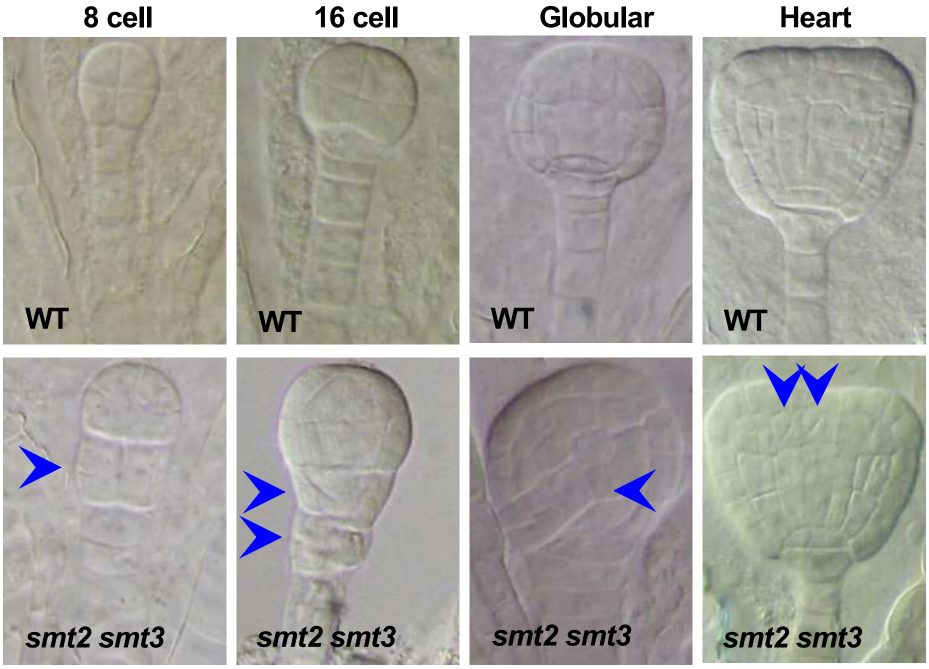

Figure S6. Defected cell division in *smt2 smt3* embryos.

Arrow heads indicate the sites of abnormal cell division.

Figure S7

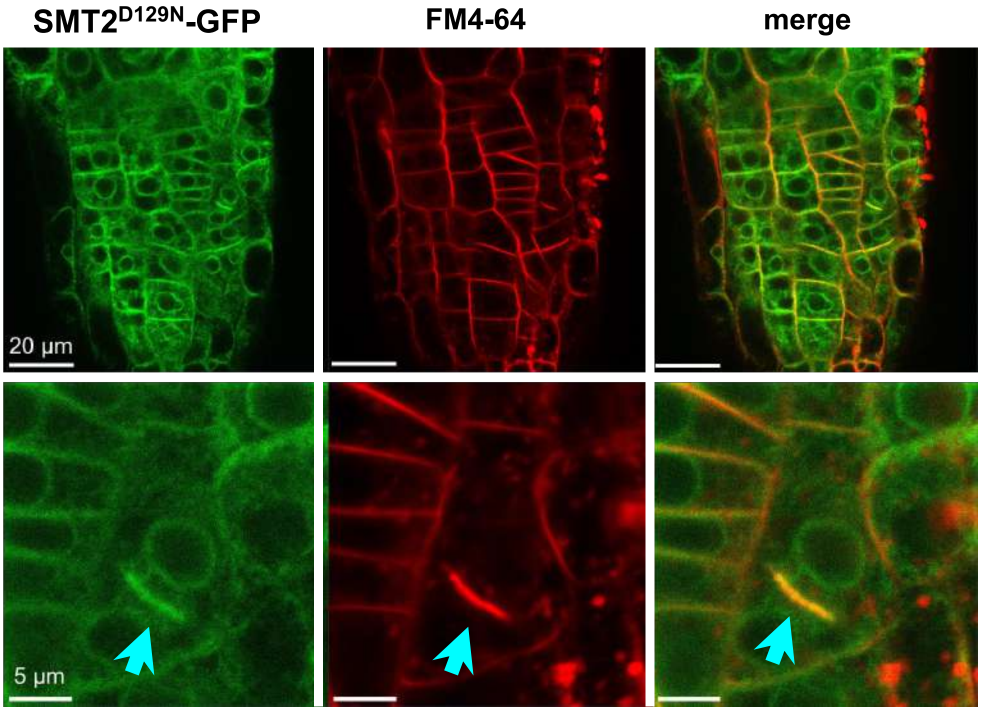

Figure S7. ***smt2 smt3*** **root tissues expressing SMT2^{D129N}-GFP.**

proSMT2::SMT2^{D129N}-GFP was used to transform +/*smt2*;*smt3/smt3*. *smt2 smt3* double mutant lines expressing SMT2^{D129N}-GFP were selected from the progeny of the self-pollination of the mutant line (+/*smt2*;*smt3/smt3*). The tissues were stained with FM4-64 for the PM visualization. Arrows indicate the abnormal cell plate.

Figure S8A

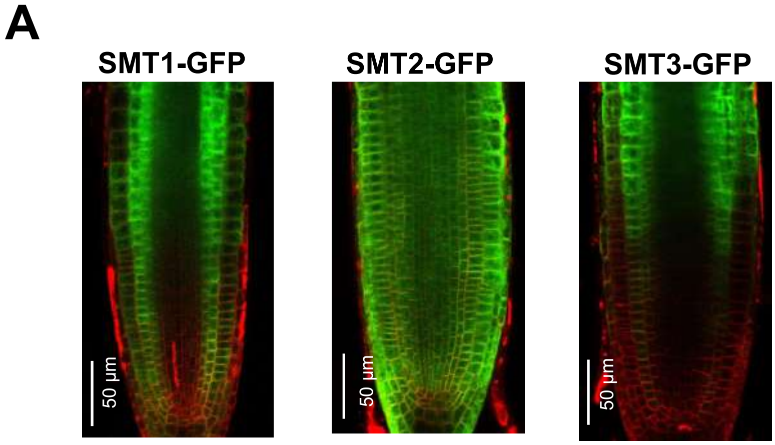

Figure S8A. Expression patterns of SMT1-GFP, SMT2-GFP, and SMT3-GFP in root tips.

WT plants were transformed using *proSMT1::SMT1-GFP*, *proSMT2::SMT2-GFP*, and *proSMT3::SMT3-GFP.* Three-day-old seedlings were used for the confocal microscopic analysis.

Figure S8B

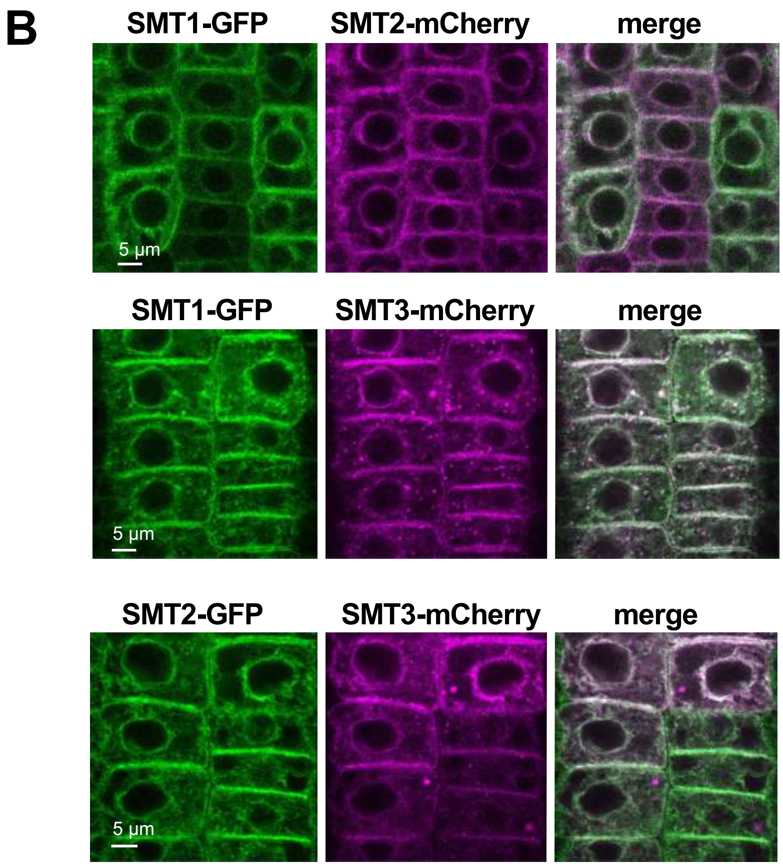

Figure S8B. Colocalization of SMT1, SMT2, and SMT3.

SMT1-GFP and SMT2-GFP expression lines were crossed with SMT2-mCherry and SMT3-mCherry expression lines, respecitivey. Three-day-old seedlings were used for the confocal microscopic analysis.

Figure S9

A

B

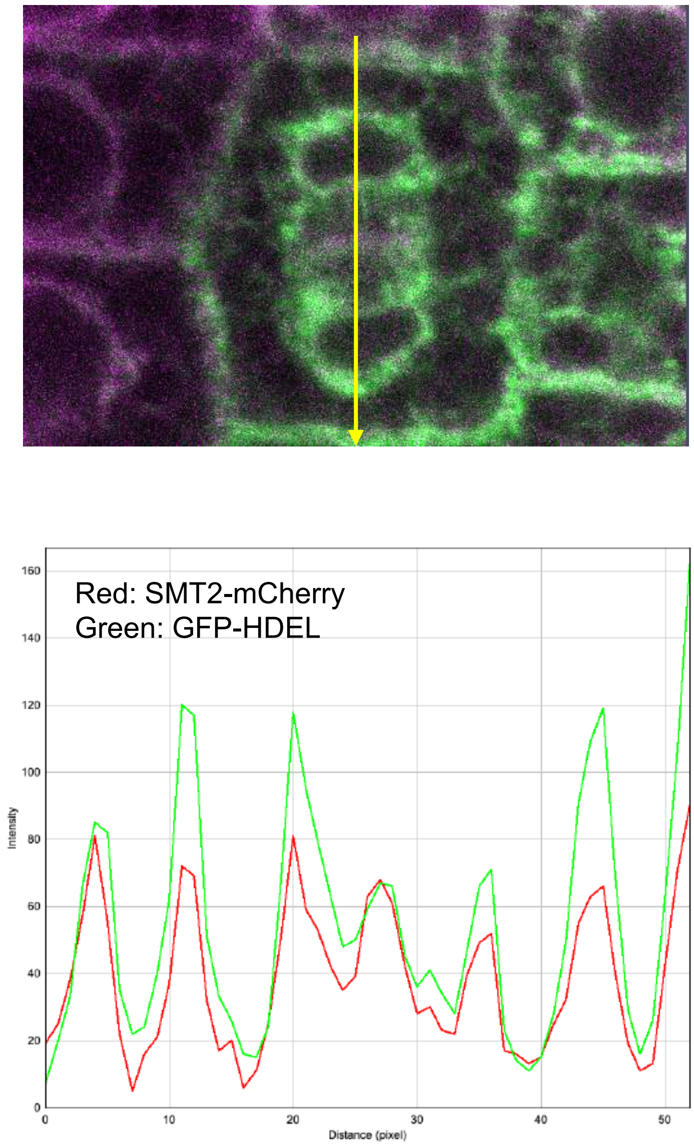

Figure S9. Colocalization of SMT2-mGFP and GFP-HDEL during cell division.

(A) An example of colocalization of SMT2 with GFP-HDEL is shown using the same image shown in Figure 1B. Fluorescence intensity plot along the indicated line by the yellow arrow. The plot was obtained using the RGB Profile Plot in the ImageJ software.

(B) The signal intensities from SMT2-mCherry (red line) and GFP-HDEL (green line) were highly correlated.

Figure S10

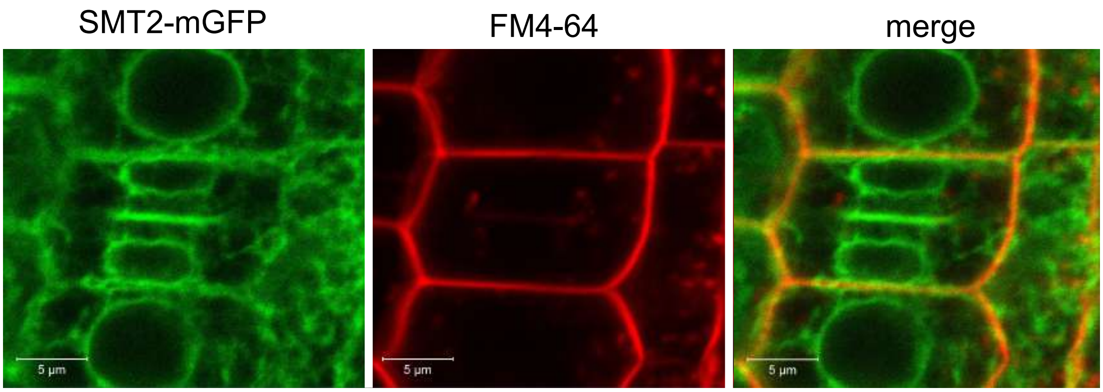

Figure S10. Colocalization of SMT2-mGFP and FM4-64 at the division plane.

Roots of 3-day-old seedlings of the SMT2-mGFP expression line was stained with FM4-64. The vesicles stained by FM4-64 did not overlap with SMT2-mGFP in the cytosolic regions but colocalized at the division plane.

Figure S11

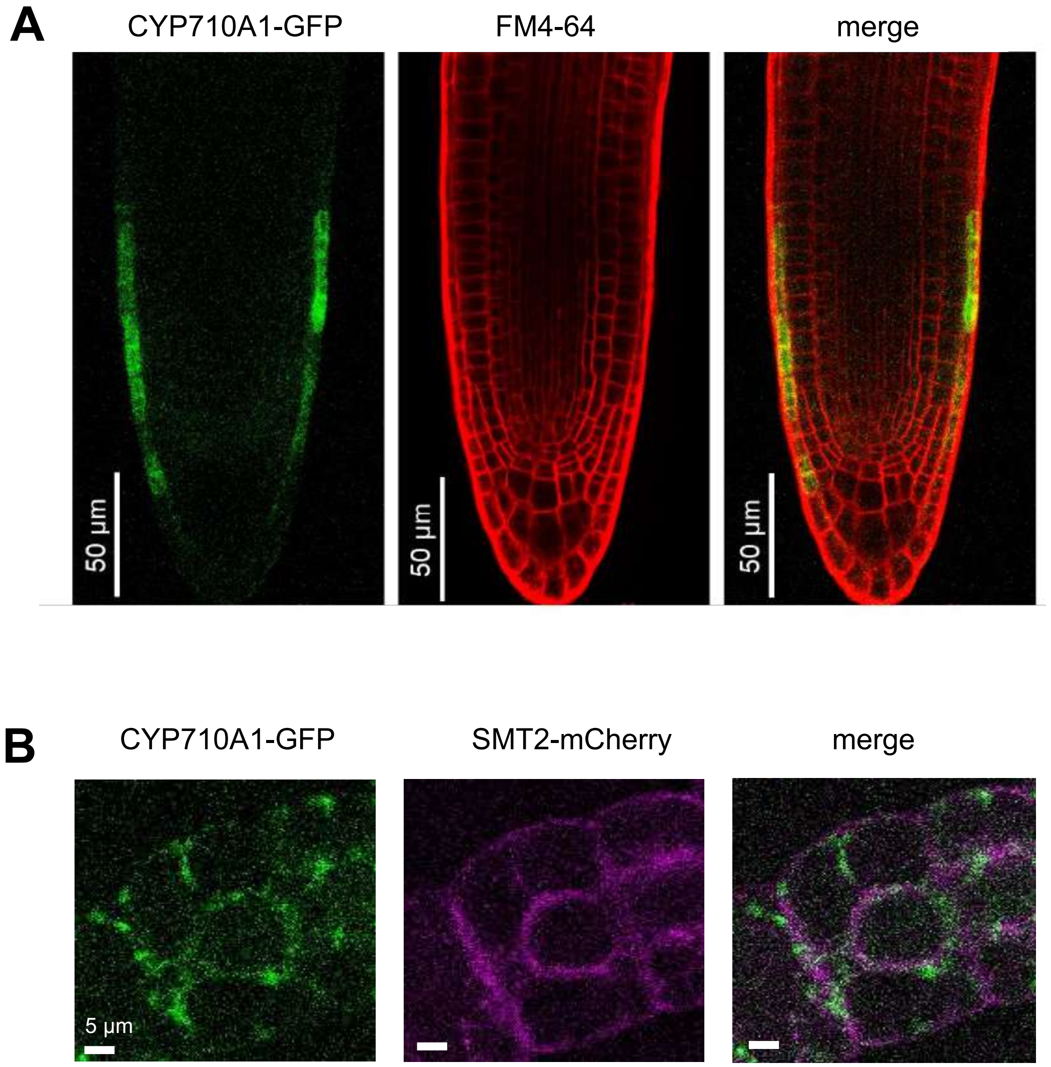

Figure S10. Expression of CYP710A1.

(A) *proCYP710A1:: CYP710A1-GFP* was used to transform Arabidopsis WT plants. Three-day-old seedlings of the CYP710A1 expression line were subjected to FM4-64 staining.

(B) Tobacco BY2 cells were transformed using both *proCYP710A1::CYP710A1-GFP and proSMT2::SMT2-mCherry*.

Figure S12

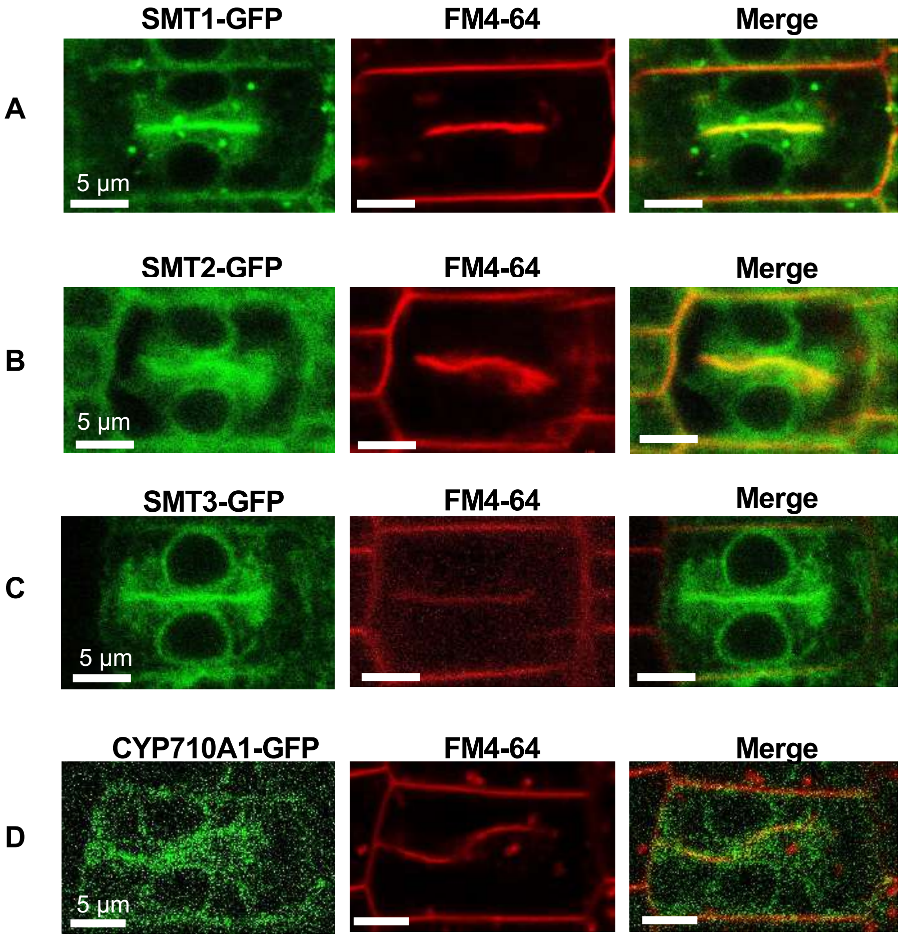

Figure S12. Colocalization of sterol biosynthetic enzymes at division plane.

Fluorescent fusion proteins, SMT1-GFP (A), SMT2-GFP (B), SMT3-GFP (C), and CYP710A1-GFP (D), were expressed in Arabidopsis WT plans under the control of the individual promoters. Three-day-old seedlings were stained using FM4-64 for the PM visualization.

Figure S13

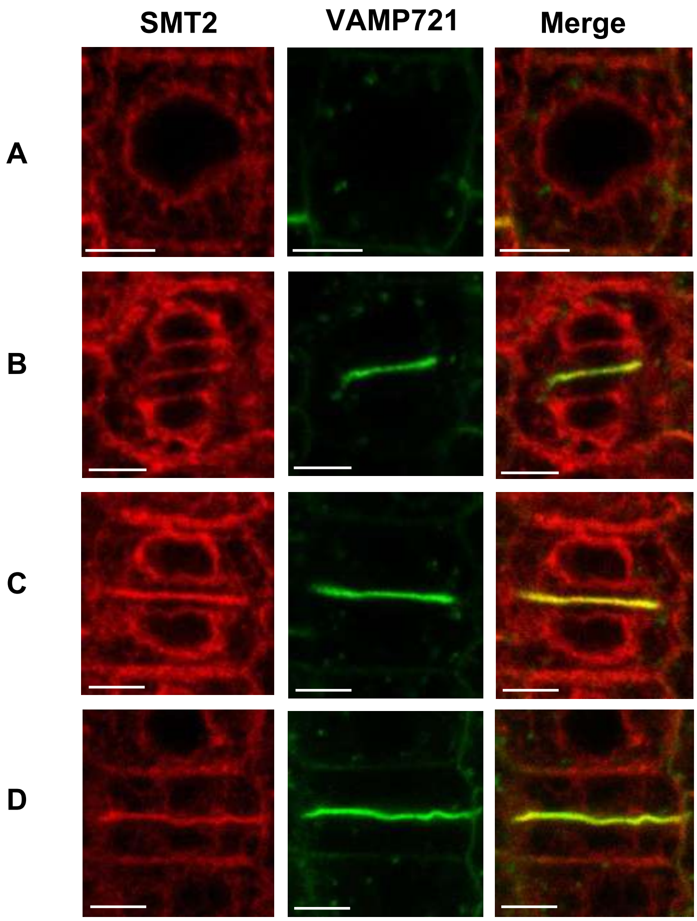

Figure S13. Colocalization of SMT2-mCherry and VAMP721.

GFP-VAMP721 expressing [39, 40] was crossed with WT expressing *SMT2-mCherry.* Three-day-old seedlings from F3 progeny were subjected to the ClearSee treatment [43] for confocal microscopic analysis. SMT2-mCherry and GFP-VAMP colocalized at the cell plate, while no SMT2-mCherry signal overlapped with the VAMP721 vesicles appeared in the cytoplasmic regions. Scale bars = 5 μm.

Figure S14

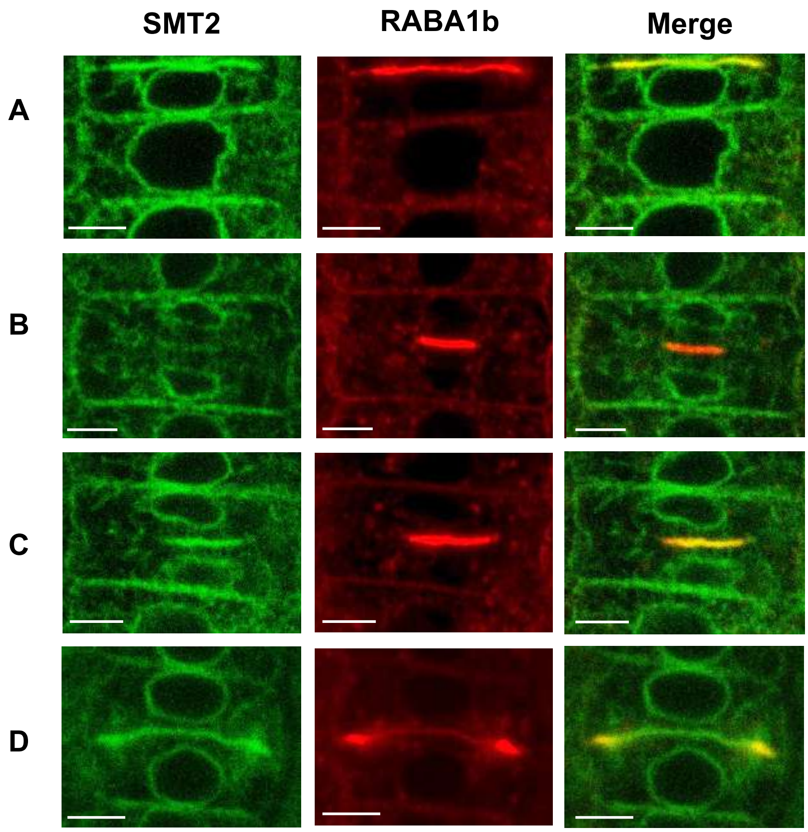

Figure S14. Colocalization of SMT2-mCherry and RABA1b.

RFP-RABA1b expressing [41] was crossed with WT expressing SMT2-GFP. Three-day-old seedlings of F3 progeny were used for confocal microscopic analysis. SMT2-GFP and RFP-RABA1b colocalized at the cell plate, while no SMT2-GFP signal overlapped with the RFP-RABA1b vesicles appeared in the cytoplasmic regions. Scale bars = 5 μm.

Figure S15A

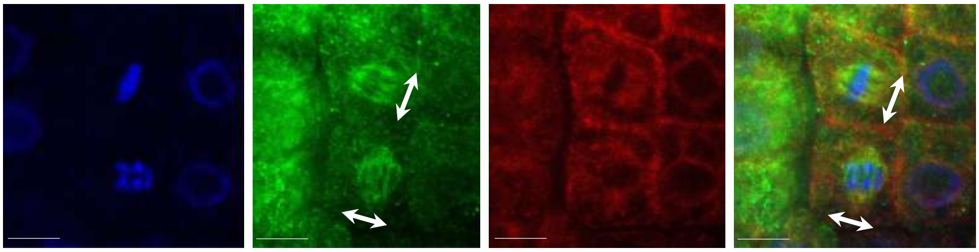

Figure S15A. Immunohistochemical analysis of the localization of SMT2-mGFP and cortical microtubules in WT.

Whole-mount immunolabeling experiments were performed to compare the organization of α-tubulin in WT plants expressing *proSMT2::SMT2-mGFP*. Antibodies were used at following dilutions 1:2000; mouse anti-a-tubulin IgG (Invitrogen), 1:500; rabbit anti-GFP (Sigma), 1:1000; Alexa Fluor 488 goat anti-rabbit IgG (Invitrogen), 1:500; Alexa Fluor 568 goat anti-mouse IgG (Invitrogen), 1:500; Alexa Fluor 568 goat anti-mouse IgG (Invitrogen). The samples were stained with 10 mg/ml 4',6-diamidino-2-phenylindole (DAPI). scale bar: 5 µm

Figure S15B

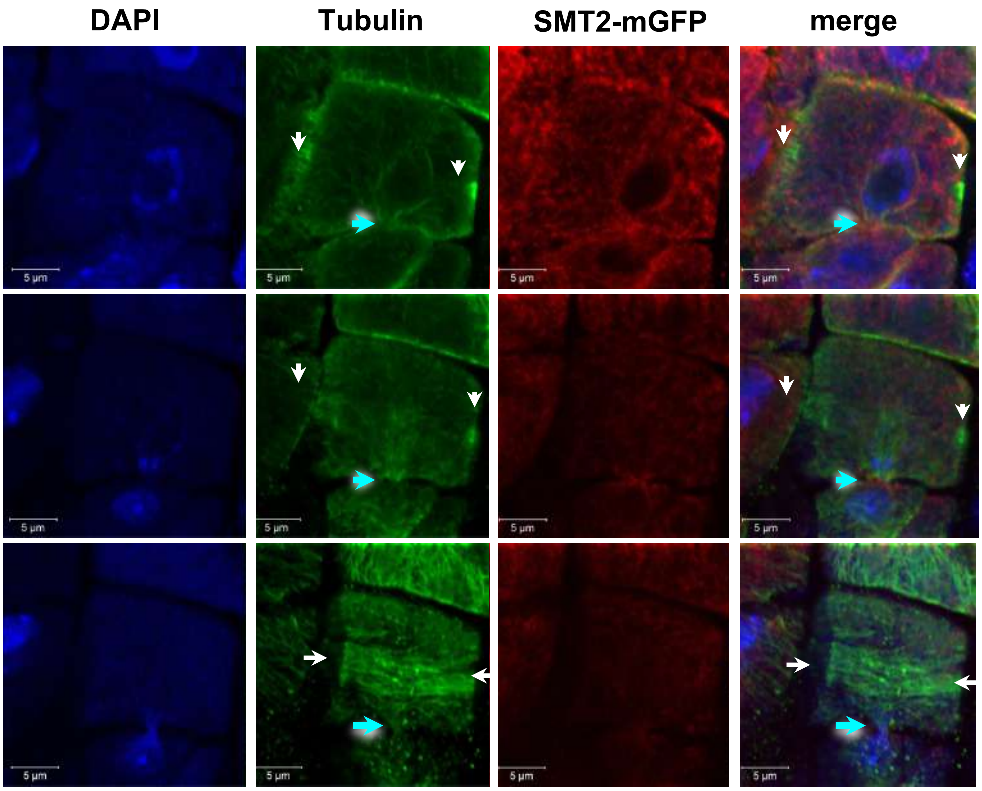

Figure S15B. Immunohistochemical analysis of the localization of SMT2-mGFP and cortical microtubules in WT.

Whole-mount immunolabeling experiments were performed to compare the organization of α-tubulin in WT plants expressing *proSMT2::SMT2-mGFP*. Antibodies were used at following dilutions 1:2000; mouse anti-a-tubulin IgG (Invitrogen), 1:500; rabbit anti-GFP (Sigma), 1:1000; Alexa Fluor 488 goat anti-rabbit IgG (Invitrogen), 1:500; Alexa Fluor 568 goat anti-mouse IgG (Invitrogen), 1:500; Alexa Fluor 568 goat anti-mouse IgG (Invitrogen). The samples were stained with 10 mg/ml 4',6-diamidino-2-phenylindole (DAPI). scale bar: 5 µm

Figure S16

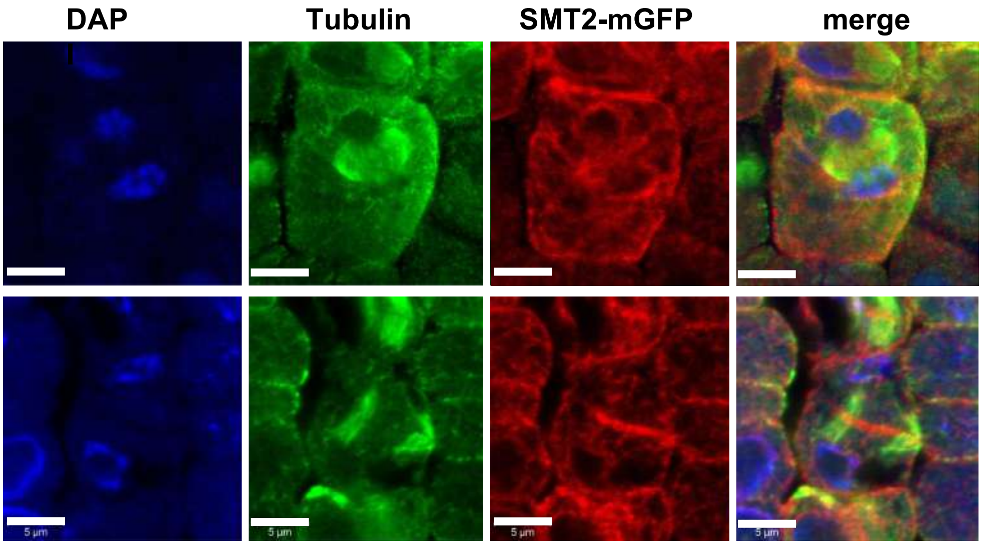

Figure S16. Colocalization of SMT2-mGFP and cortical microtubules in *smt2 smt3*.

Whole-mount immunolabeling experiments were performed to compare the organization of α-tubulin in *smt2 smt3* plants expressing D129N-mGFP. Antibodies were used at following dilutions 1:2000; mouse anti-a-tubulin IgG (Invitrogen), 1:500; rabbit anti-GFP (Sigma), 1:1000; Alexa Fluor 488 goat anti-rabbit IgG (Invitrogen), 1:500; Alexa Fluor 568 goat anti-mouse IgG (Invitrogen), 1:500; Alexa Fluor 568 goat anti-mouse IgG (Invitrogen). The samples were stained with 10 mg/ml 4',6-diamidino-2-phenylindole (DAPI). scale bar: 5 μm

Figure S17

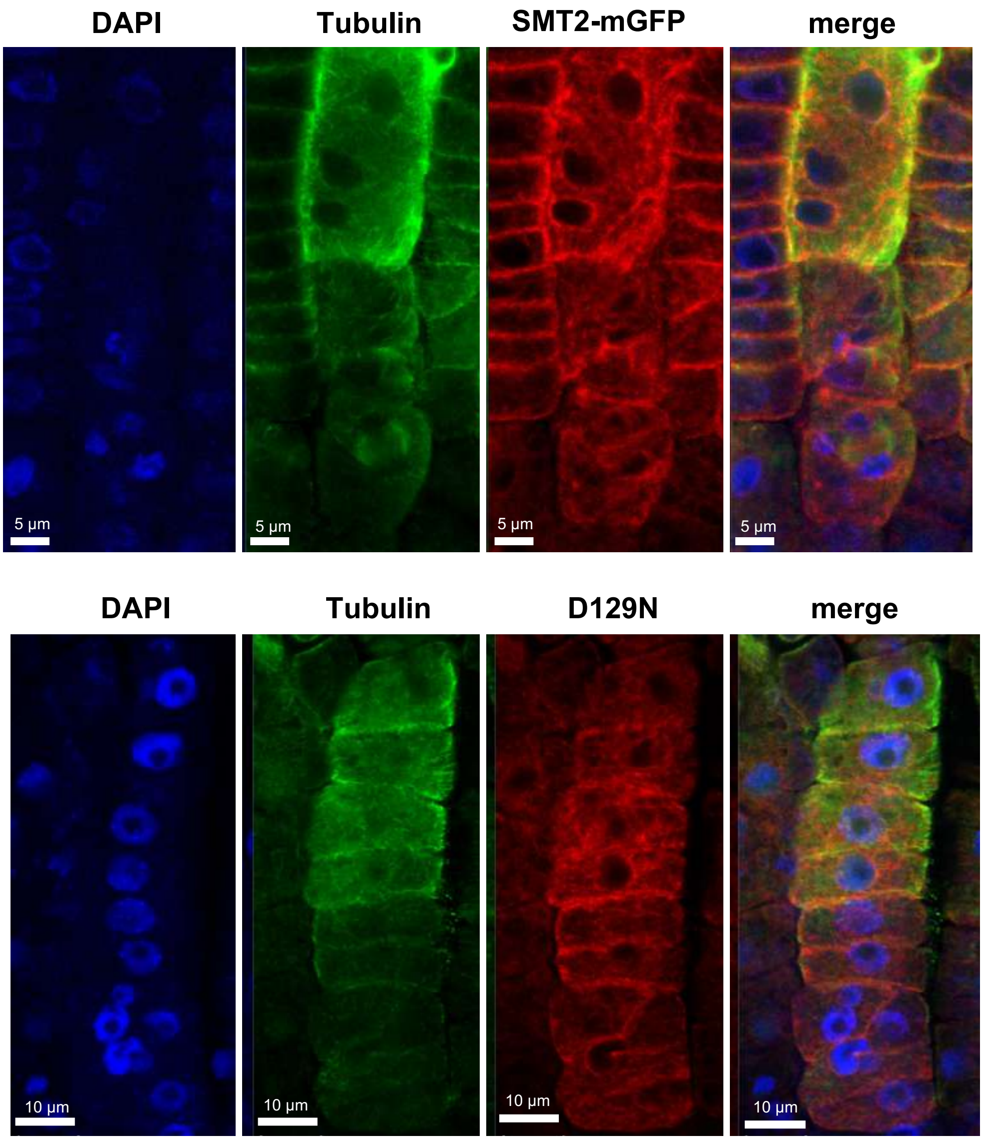

Figure S17 Organization of α-tubulin in *smt2 smt3* plants expressing D129N-mGFP.

Whole-mount immunolabeling experiments were preformed using antibodies at following dilutions 1:2000; mouse anti-a-tubulin IgG (Invitrogen), 1:500; rabbit anti-GFP (Sigma), 1:1000; Alexa Fluor 488 goat anti-rabbit IgG (Invitrogen), 1:500; Alexa Fluor 568 goat anti-mouse IgG (Invitrogen), 1:500; Alexa Fluor 568 goat anti-mouse IgG (Invitrogen). The samples were stained with 10 mg/ml 4',6-diamidino-2-phenylindole (DAPI). scale bar: 5 µm

Figure S18

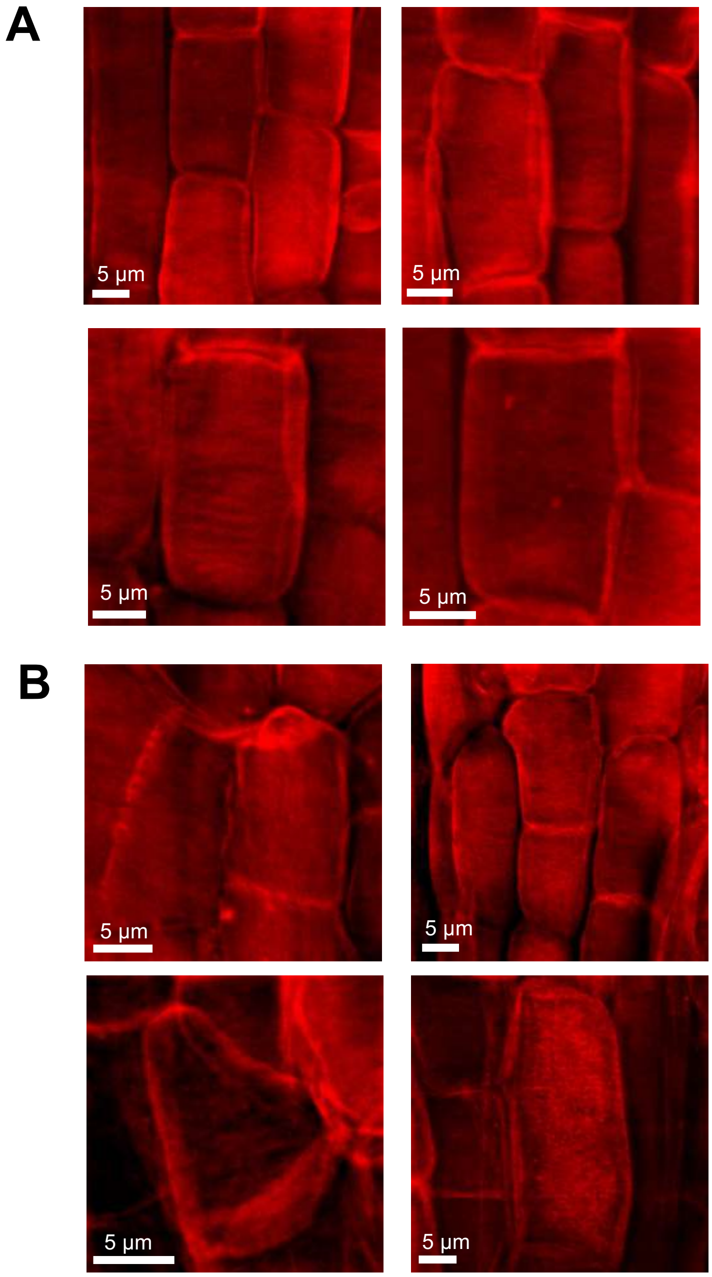

Figure S18. Cellulose microfibril structures on the cell cortical regions of WT (A) and *smt2 smt3* (B).

For cellulose staining, tissues were treated with 100 µg/mL Direct Red 23 (Sigma Aldrich Japan, Tokyo, Japan),
