# Supplementary material for: Characterization of Subcellular Dynamics of Sterol Methyltransferases Clarifies Defective Cell Division in smt2 smt3, a C-24 Ethyl Sterol-Deficient Mutant of Arabidopsis
Source: Biomolecules. 2024 Jul 19;14(7):868. doi: 10.3390/biom14070868 (PMC11275053; doi:10.3390/biom14070868)
Supplement: Supplementary file 1 [file biomolecules-14-00868-s001.zip › Supplementary Tables.pdf]

Table S1. Summary of sterol biosynthetic enzymes expressed as fusion proteins with fluorescent proteins.

Expressed enzyme	fusion protein*
SMT1-GFP	sterolmethyltransferase1 fused with GFP
SMT1-mGFP**	sterolmethyltransferase1 fused with mGFP
SMT2-GFP	sterolmethyltransferase2 fused with GFP
SMT2-mGFP**	sterolmethyltransferase2 fused with mGFP
SMT2-mCherry	sterolmethyltransferase2 fused with mCherry
$SMT2^{D129N}$-mGFP***	sterolmethyltransferase2 ($SMT2^{D129N}$) with mGFP
$SMT2^{D129N}$-mCherry***	sterolmethyltransferase2 ($SMT2^{D129N}$) with mCherry
SMT3-mGFP**	sterolmethyltransferase3 fused with mGFP
SMT3-mCherry	sterolmethyltransferase3 fused with mCherry
CYP710A1-GFP	CYP710A1 (sterol C-22 desaturase) fused with GFP

Sterol biosynthetic enzymes, SMT1 (AT5G13710), SMT2 (AT1G20330), SMT3 (AT1G76090), and CYP710A1 (AT2G34500), were fused with fluorescent proteins at their C-terminal ends. These fusion proteins were expressed under the control of the promoters of their corresponding genes.

*Fluorescent proteins were fused with the C-terminal ends of the enzyme proteins.
**mGFP is a GFP variant with a point mutation of ^{206}Ala to ^{206}Lys. In this study, differences in the subcellular localizations of GFP and mGFP were not apparent.
*** A point mutation (D129N) was introduced to convert ^{129}Asp to ^{129}Asn in the SAM binding site of SMT2 protein and expressed as fusion proteins with mGFP (SMT2D129N-mGFP) and mCherry (SMT2D129N-mCherry). $SMT2^{D129N}$ was designed to generate a catalytically inactive form in reference to a previous report [36].

Table S2. Sterol compositions of WT, *smt2 smt3*, and *smt2 smt3* expressing SMT2-GFP.

sterol (in %)	*peak number	wt	*smt2 smt3*	**CDM
cycloartenol	1	2.26	***nd	2.24
24-methylenecycloartenol	2	2.88	2.36	3.99
cycloeucalenol	3	0.5	nd	0.8
cholesterol	4	1.67	2.32	1.63
brassicasterol	5	3.21	6.46	3.1
24-methylenecholesterol	6	6.13	nd	5.1
campesterol		23.09	87.5	24.14
isofucosterol	7	19.36	nd	10.1
sitosterol		39.29	1.21	45.18
stigmasterol	8	1.29	0.5	3.5

*peak number; corresponds the peaks shown in Figure S2,

**CDM; Complemented Double Mutant *smt2 smt3* expressing *proSMT2::SMT2-GFP*,

***nd: not detected.

Table S3. Expression plasmids of sterolmethyltransferase-fluorescent protein fusions.

Expression cassette	vector	Transformed plant	Analyzed plant
proSMT1::SMT1-EGFP	pBIN plus	*A. thaliana* Col-0 (WT)	T_3
		A. thaliana smt2 smt3	T_3
proSMT2::SMT2-mGFP	pBIN plus	*A. thaliana* Col-0 (WT)	T_3
proSMT2::SMT2-mCherry	pBI101	*A. thaliana smt2 smt3*	T_3
proSMT2::D129N-mGFP	pBIN plus	*A. thaliana smt2 smt3*	T_3
proSMT2::D129N-mCherry	pBI101	*A. thaliana smt2 smt3*	T_2
pro35S::sGFP-Tubulin	pBI121	*A. thaliana* Col-0 (WT)	T_4
		A. thaliana smt2 smt3	T_3

Table S4. Primer list.

Target fragment	primer name	5’ → 3’
SMT1 promoter	pSMT1 Fw	AAGCTTTATGTAGACACACCCCTACACAA
	pSMT1 Rv	GGTACCAGCACTTATGCAACAACGGAC
SMT1 CDS*	SMT1-Fw	AAGCTTATGGATCTCGCGTCGAATCTT
	SMT1-Rv	GGTACCTCATCCGGACTCTGGCTTGGCCCGG
SMT1 CDS with a linker	SMT1-linker Rv	GGATCCGCTGCCTCCTGCAGCGGCCGCTCCGG ACTCTGGCTTCCGGGCC
SMT2 promoter	pSMT2 Fw_N	AGTACTGTTATGGTCTGATTAGAA
	pSMT2 Rv_XbaI	TCTAGATTAAGAGTGAGGAAGACCAAGAG
SMT2 promoter and SMT2 CDS with a linker	pSMT2_Fw2	TTTAAGCTTTCTGGTCGGTACTTCTCTCTC
	SMT2_cDNA_Rv_ linker	TTTGGATCCGCTGCCTCCTGCAGCGGCCGCTC CGGAAGAACTCTCCTCCG GTGA
SMT2 CDS	BamHI_SMT2_FW	GGATCCATGGACTCTTTAACACTCTTCTTC
	SMT2_EcoRI_RV	GAATTCTCAAGAACTCTCCTCCGGTG
SMT3 promoter	pSMT3Fw_N	AGTACTCTTCGAATATAACAGCTGGAGATG
	pSMT3 Rv_XbaI	TCTAGAACAGGGAGAAAGAGAGAAGCA
SMT3-linker	SMT3_mCherry_FW	AAATCTAGAATGGACTCGGTGGCTCT
	SMT3_linker2_BamHI	TTTGGATCCGCTGCCTCCTGCAGCGGCCGCTC CGGATTCAGAAGCTTTCTCTGGTT
SMT3 CDS	BamHI_SMT3_FW	AAAGGATCCATGGACTCGGTGGCTCTCT
	SMT3_SacRI_RV	GAGCTCTCATTCAGAAGCTTTCTCTGGTTT
CYP710A1 promoter	proAt710A1 Fw	GTCGACTGGAAGTCTTCGAGACTGAA
	proAt710A1 Rv	GGTACCGTTTCTTTGTTTCTAGCTTGTC
CYP710A1 CDS with a linker	At710A1 Fw	GGTACCATGGTTTTCTCTGTTTCTATATTT
	At710A1 linker Rv	TCTAGAGCTGCCTCCTGCAGCGGCCGCTCCGG AGGAAAAGTTGGGATATTTGCG
mCherry CDS	mCherry_Fw	AAAGGATCCATGGTGAGCAAGGGCGA
	mCherry/Rv	TTGAGCTCTTACTTGTACAGCTCGTCCATG
D129N point mutation	SMT2-*Sac* I	GAGCTCTGTTCACCTGATACT
	SMT2-*Sca* I	AGTACTGGTCTTTCTTCCGCC
	mGFP_A206K Fw	CAGTCCAAACTGAGCAAAGACCCCAAC

mGFP point mutation	mGFP_A206K Rv	GCTCAGTTGGACTGGGTGCTCAGGTA

*CDS: coding sequence
